# Inferring allele-specific copy number aberrations and tumor phylogeography from spatially resolved transcriptomics

**DOI:** 10.1038/s41592-024-02438-9

**Published:** 2024-10-30

**Authors:** Cong Ma, Metin Balaban, Jingxian Liu, Siqi Chen, Michael J. Wilson, Christopher H. Sun, Li Ding, Benjamin J. Raphael

**Affiliations:** 1 Department of Computer Science, Princeton University, Princeton, NJ USA; 2 Department of Medicine, Washington University in St. Louis, St. Louis, MO USA; 3 McDonnell Genome Institute, Washington University in St. Louis, St. Louis, MO USA; 4 Department of Astrophysical Sciences, Princeton University, Princeton, NJ USA; 5 Lewis-Sigler Institute for Integrative Genomics, Princeton University, Princeton, NJ USA; 6 Siteman Cancer Center, Washington University in St. Louis, St. Louis, MO USA; 7 Department of Genetics, Washington University in St. Louis, St. Louis, MO USA

**Keywords:** Computational biology and bioinformatics, Phylogeny

## Abstract

Analyzing somatic evolution within a tumor over time and across space is a key challenge in cancer research. Spatially resolved transcriptomics (SRT) measures gene expression at thousands of spatial locations in a tumor, but does not directly reveal genomic aberrations. We introduce CalicoST, an algorithm to simultaneously infer allele-specific copy number aberrations (CNAs) and reconstruct spatial tumor evolution, or phylogeography, from SRT data. CalicoST identifies important classes of CNAs—including copy-neutral loss of heterozygosity and mirrored subclonal CNAs—that are invisible to total copy number analysis. Using nine patients’ data from the Human Tumor Atlas Network, CalicoST achieves an average accuracy of 86%, approximately 21% higher than existing methods. CalicoST reconstructs a tumor phylogeography in three-dimensional space for two patients with multiple adjacent slices. CalicoST analysis of multiple SRT slices from a cancerous prostate organ reveals mirrored subclonal CNAs on the two sides of the prostate, forming a bifurcating phylogeography in both genetic and physical space.

## Abstract (web summary)

This work presents CalicoST for inferring allele-specific copy numbers and reconstructing spatial tumor evolution by using spatial transcriptomics data.

## Main

Tumors evolve through acquisition of somatic mutations—including single nucleotide variations (SNVs), CNAs and large-scale structural variations. Sequencing of somatic mutations in bulk tumors^1,2^ or disassociated single cells^3–5^ has revealed the genetic heterogeneity within tumors and enabled the reconstruction of a tumor’s evolutionary history^6–8^. At the same time, tumors exhibit heterogeneity and undergo evolution within physical space, expanding and regressing based on interactions with other cells and the local microenvironment. Incorporating the spatial perspective into somatic evolution studies is a key challenge^9^, but has been hampered by a lack of spatial data.

Recent technological advances in spatial sequencing provide a promising direction for studies of spatiotemporal tumor evolution. While high-quality spatial DNA sequencing would provide the ideal dataset for spatiotemporal evolution studies, such technologies remain in active development^10^ and are not yet widely applied. However, SRT technologies that measure RNA simultaneously from thousands of spatial locations in a tissue have found extensive applications in analyzing the spatial organization of transcriptionally defined cell types within a tumor^11–14^. Even though somatic mutations occur in DNA and are not directly measured by SRT, large CNAs leave a signature in gene expression; namely, a deletion of a genomic region tends to result in underexpression of genes in the region, while an amplification tends to result in overexpression. Thus, identification of CNAs from transcriptomic data is a promising direction for analysis of somatic evolution in tumors.

Inferring CNAs from single-cell or spatially resolved transcriptomics is challenging as there are multiple explanations for an observed gene expression change, such as chromatin accessibility and transcription factor binding. It is typically difficult to determine whether an observed gene expression change is a result of CNAs or these other causes. Existing methods to infer CNAs from gene expression data^15–18^ assume that large CNAs alter the expression of multiple adjacent genes in a genomic region beyond that expected by other regulatory effects. However, the variability of expression is so large between genes that these methods have limited accuracy in inferring CNAs and are not robust across tissues, patients and cancer types. A few methods aim to address these challenges by combining single-cell RNA sequencing (scRNA-seq) and single-cell DNA sequencing^19,20^, but performing both types of sequencing of the same tumor is uncommon. In addition, both scRNA-seq and SRT are sparse, generally having more than 75% zero counts across genes and cells/spots. Finally, the commonly used 10x Genomics Visium^21^ and Slide-seqV2 (ref. ^22^) SRT technologies pose an additional challenge beyond scRNA-seq: they measure RNA from a mixture of cells at each spatial spot.

Importantly, a CNA alters one of the two parental chromosomes, and thus the identification of ‘allele-specific CNAs’ is essential for deriving a comprehensive description of CNAs in a tumor. For example, copy number neutral loss of heterozygosity (CNLOH)—an event where a region of one parental chromosome is deleted and the other parental chromosome is amplified so that the total copy number of the locus is unchanged—is common in cancer^23–25^. Similarly, mirrored subclonal CNAs—where different cancer cells have independent gains or losses of different parental alleles—are also common in cancer^4,26^. Importantly, neither of these events alters the total number of copies of the genomic locus in a cell. Thus, these events are invisible to total copy number analysis, which may lead to incorrect identification of tumor clones and inaccurate tumor phylogenies. Most existing methods to detect CNAs from transcriptomics data do not distinguish between alleles^15–17^, while a few recent methods for identification of allele-specific CNAs from scRNA-seq data^27–29^ are challenged by the weak signal to distinguish the two parental alleles in scRNA-seq data.

We introduce CalicoST, a method to infer allele-specific CNAs in SRT data and to use these CNAs to reconstruct spatial tumor evolution, or a phylogeography. CalicoST identifies CNLOH and mirrored subclonal CNAs that are invisible to total copy number analysis. The phylogeographic reconstruction describes the accumulation of allele-specific CNAs over time and the spread of the tumor clones across physical space. We validate CalicoST using SRT data from nine patients from the Human Tumor Atlas Network (HTAN; WashU cohort)^30^ with matched whole-exome sequencing (WES) data. CalicoST achieves at least 86% accuracy in its inferred allele-specific copy numbers, 21% higher than previous methods. We reconstruct a three-dimensional phylogeography for a patient with colorectal cancer (CRC) and a patient with breast cancer from the HTAN with multiple adjacent slices. The phylogeography reveals the spatial direction of tumor expansion in the third dimension that was not apparent from a single slice. We also apply CalicoST to multiple SRT slices from a prostate cancer patient, identifying mirrored subclonal CNAs that suggest convergent evolution in the tumor. The reconstructed phylogeography partitions cancer clones into the left and the right sides of the prostate, revealing the separation of the clones in both physical and genetic space. CalicoST enables the study of spatial tumor evolution, progression and metastasis, and will be helpful for further applications to cancer diagnosis and treatment.

## Results

### CalicoST algorithm

**Fig. 1:**
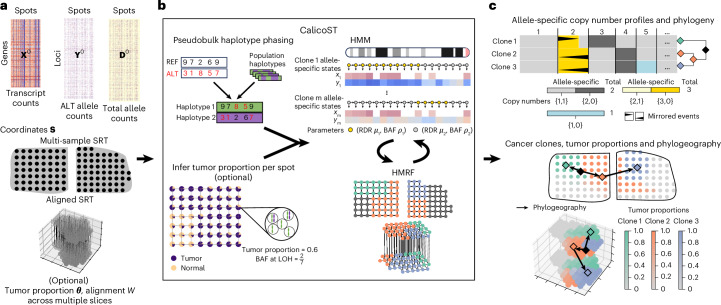
CalicoST infers allele-specific copy numbers and a phylogeography of a tumor from one or more SRT samples from the same patient. **a**, Inputs to CalicoST are transcript counts **X**^0^, allele counts **Y**^0^ and **D**^0^, spatial coordinates **S** from one or more SRT slices or a 3D alignment of slices. **b**, CalicoST phases input alleles in **Y**^0^ and **D**^0^ using a database of haplotypes. Optionally, CalicoST infers tumor proportion per spot using the BAF. CalicoST jointly models transcript counts and allele counts as functions of allele-specific copy number states within each clone. CalicoST uses an HMM to model correlations between copy number states from adjacent genomic regions and a HMRF to model correlations between the cancer clones assigned to neighboring spatial locations. **c**, CalicoST infers allele-specific integer copy numbers for one or more cancer clones, a phylogeny relating these clones, a clone label, an optional tumor proportion for each spot and a phylogeographic model of the spatial expansion of cancer clones.

CalicoST infers allele-specific CNAs and a phylogeographic reconstruction of tumor evolution from one or more SRT samples from a tumor (Fig. 1). CalicoST has the following key features: (1) Identifies allele-specific integer copy numbers of transcribed regions for each cancer clone, revealing events such as CNLOH and mirrored subclonal CNAs that are invisible to total copy number analysis. (2) Assigns each spot a clone label indicating the allele-specific copy number profiles of the spot. (3) Infers a phylogeny relating the identified copy number profiles (cancer clones) as well as a phylogeography that combines somatic evolution and spatial dissemination of clones. (4) Infers and models the normal cell admixture in SRT technologies that are not single-cell resolution (for example, 10x Genomics Visium) to infer more accurate allele-specific copy numbers and cancer clones. (5) Simultaneously analyzes multiple regional or aligned SRT slices from the same tumor.

The inputs to CalicoST are a spatial coordinate matrix **S**, a transcript count matrix **X**^0^ whose entries are the total number of reads from each transcript in each spot, an allele count matrix **Y**^0^ whose entries are the number of reads from the non-reference allele of germline heterozygous single nucleotide polymorphisms (SNPs), and total allele count matrix **D**^0^ whose entries are the total number of reads from both reference and non-reference alleles of the same set of germline heterozygous SNPs as **Y**^0^ (Fig. 1a). The matrix **X**^0^ is readily obtained from standard SRT analysis pipelines, while the matrices **Y**^0^ and **D**^0^ are calculated from a specialized pipeline that counts the reference and non-reference alleles at known locations of germline SNPs. The counts at individual SNPs are sparse: 98.8% of SNP loci have zero total count within each individual spot and another 0.9% have only one total count. To reduce the sparsity, CalicoST phases SNPs using both a database of haplotypes and the allele counts in the pseudobulk of spots, and then for each spot aggregates transcript counts and allele counts from the same haplotype across adjacent genomic loci to obtain the matrices **X**, **Y** and **D**.

In addition, some SRT technologies (for example, 10x Genomics Visium), may lack single-cell resolution, measuring multiple cells within each spatial spot. This admixture dilutes the signal for the identification of CNAs and cancer clones. Optionally, CalicoST infers the tumor proportion ***θ*** for each spot according to the B allele frequency (BAF) in genomic regions with loss of heterozygosity (LOH) in the tumor, and further uses ***θ*** to infer the full allele-specific copy number profiles.

The core of CalicoST is a generative probabilistic model of the observed variables **X**, **Y** as a function of the unobserved allele-specific copy numbers and clone labels ***ℓ*** given **S** and **D**. Individual entries in **X** and **Y** provide poor estimates of the allele-specific copy number due to low sequence coverage and confounding by other sources of variation, such as variable gene expression. Thus, CalicoST models the correlations between copy numbers from multiple adjacent loci in the genome using a hidden Markov model (HMM) and the correlations between clone labels ***ℓ*** from adjacent spots using a hidden Markov random field (HMRF), assuming adjacent spots are likely to be genetically similar.

Finally, CalicoST reconstructs a phylogeographic model to describe the ancestral relationships between the inferred clones as well as the spatial location of the ancestors of these clones (Fig. 1c). Constructing a phylogeny from copy number profiles is challenging and typically requires complicated evolutionary models^31,32^. CalicoST infers a phylogeny of cancer clones using LOH events, which have the important property of being irreversible; that is, once a parental haplotype is lost in a lineage, it cannot be regained. CalicoST constructs a tumor phylogeny using the star homoplasy model implemented in Startle^33^, and infers the spatial location of ancestral clones using a diffusion model.

### CalicoST infers accurate allele-specific CNAs in HTAN

We applied CalicoST to infer allele-specific copy numbers on 10x Genomics Visium Spatial Transcriptomics data from 12 patients (26 slices) in HTAN (WashU cohort)^30^ across three cancer types (‘Running CalicoST on SRT data’). For 11 of these patients, WES data from adjacent bulk tumor sections were also available, and we derived allele-specific integer copy numbers for 9 of these patients with sufficient tumor purity using HATCHet2 (ref. ^34^; Supplementary Text 11). We used these copy numbers as the ground truth to benchmark the inferred CNAs from SRT data by CalicoST. We estimated the tumor purity per spot using allele frequency signals in LOH regions predicted by CalicoST (‘Inferring tumor proportions using BAF’) and explicitly modeled the inferred tumor proportions in CNA inference (Supplementary Text 3).

**Fig. 2:**
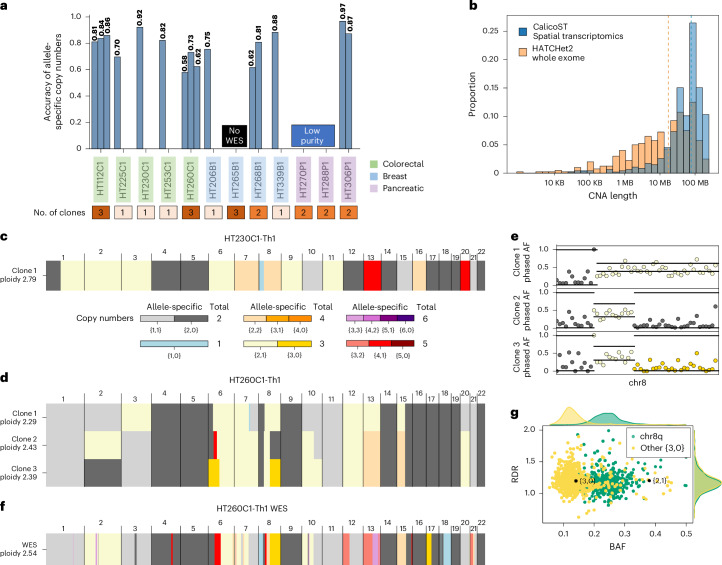
CalicoST infers accurate allele-specific copy numbers in HTAN samples. **a**, Accuracy of allele-specific copy numbers across 12 patients from HTAN (WashU cohort) inferred by CalicoST. Each bar represents an inferred cancer clone. **b**, Length distribution of CNAs identified by CalicoST from SRT data and identified by HATCHet2 from WES for the 9 patients with matched WES data of sufficient tumor purity. Blue bars are CalicoST, and orange bars are HATCHet2, with gray bars indicating the overlap of the two histograms. The median length is 77.4 Mb for CalicoST and 30 Mb for HATCHet2 (vertical dashed lines). **c**, Allele-specific integer copy numbers inferred by CalicoST from SRT data from a patient with CRC liver metastasis (HT230C1). Rows are cancer clones, and columns are genomic bins. Colors indicate allele-specific copy numbers. **d**, Allele-specific integer copy numbers inferred by CalicoST from SRT data from a patient with CRC liver metastasis (HT260C1). **e**, Observed RDR and BAF for chr8 of HT260C1. Points are colored by the inferred allele-specific copy numbers. Horizontal black lines indicate the RDR and BAF of the corresponding copy number states estimated by the HMM. **f**, Allele-specific integer copy numbers inferred by HATCHet2 from WES data of patient HT260C1. **g**, RDR and BAF values from WES data for bins from chromosome 8q and bins from other genomic regions with a value of {3,0} copy number state. Black points are expected RDR and BAF values for {3,0} and {2,1} states from HATCHet2 analysis.

Across the 9 patients whose matched WES sample had sufficient tumor purity, the best-matching CalicoST cancer clone had 86% accuracy on average (minimum 68% and maximum 97%; Fig. 2a), and an average of 95% precision and 90% recall in the prediction of genome bins with abnormal copy number (Supplementary Text 12), respectively (Supplementary Fig. 1a,b). The median length of CNA events detected by CalicoST was 77.4 Mb, often spanning entire chromosomes (Fig. 2b), which is of a lower resolution than CNA detected by HATCHet2 on WES samples. Nevertheless, CalicoST identified CNA events as small as 1 Mb for regions with high coverage.

We evaluated the tumor proportions of spots inferred by CalicoST in three ways. First, we observed that CalicoST-inferred tumor proportions agree visually with the hematoxylin and eosin (H&E)-stained images (Extended Data Fig. 3 and Supplementary Figs. 2 and 3). Second, we compared CalicoST-inferred tumor proportions to the annotations of tumor/normal labels per spot, which were obtained by independent review of the H&E-stained images^30^, obtaining an average area under the curve (AUC) of 0.85 (Supplementary Table 5), with lower AUC for two patients (HT306P1 and HT253C1) with smaller allele counts within LOH regions. Third, we compared the tumor proportions inferred by CalicoST to proportions estimated by RCTD^35^, obtaining an average Pearson correlation of 0.76 (Supplementary Fig. 4). This is a notable agreement because RCTD requires a matched scRNA-seq to compute cell-type proportions at individual spots, while CalicoST uses only SRT data.

**Extended Data Fig. 1:**
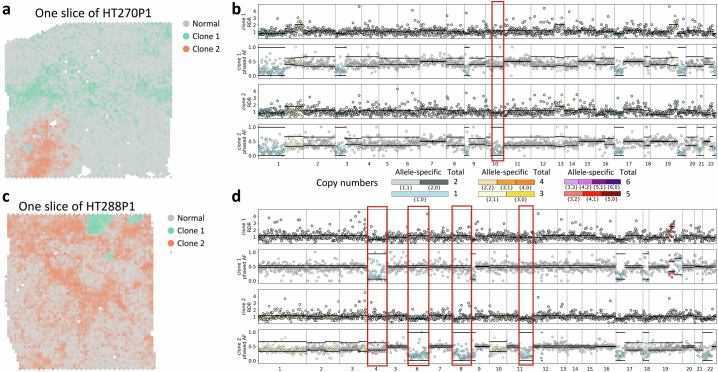
CalicoST identifies multiple clones in two PDAC patients with high degrees of normal cell infiltration. (**a**) CalicoST-inferred cancer clones in PDAC patient HT270P1. Grayscale indicates the inferred tumor proportion within each spot, where more gray indicates a higher proportion of normal cells (lower tumor proportion). (**b**) RDR and BAF along the genome for each inferred clone in HT270P1. Each point represents a genomic bin and is colored by CalicoST-inferred allele-specific copy numbers. The red box highlights a unique deletion in clone 2. (**c**–**d**) Corresponding plots for PDAC patient HT288P1. Red boxes highlight deletions that are unique to one of the inferred clones.

**Extended Data Fig. 2:**
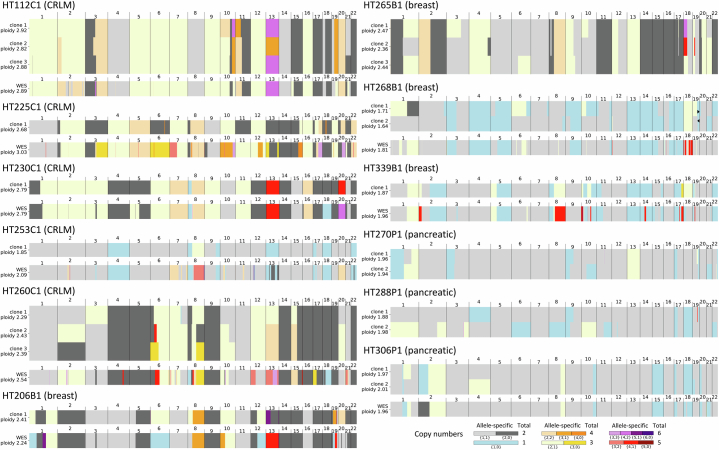
Allele-specific CNAs inferred by CalicoST from SRT data for 12 HTAN patients and by HATCHet2 from WES data. The plots for CalicoST include the allele-specific CNAs from all inferred cancer clones, labeled as ‘clone 1’, ‘clone 2’, etc. The plots for HATCHet2, labeled as ‘WES’, are included for the nine patients for whom matched WES data is available and has sufficient tumor purity.

**Extended Data Fig. 3:**
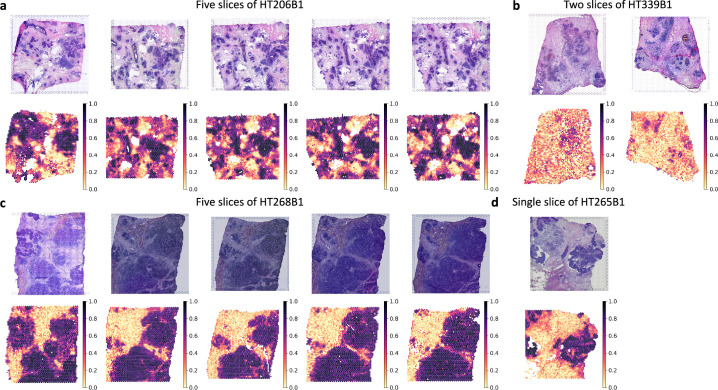
Tumor proportions inferred by CalicoST on breast cancer samples visually agree with H&E images. H&E images (top) and CalicoST-inferred tumor proportions (bottom) for breast cancer samples: (**a**) HT206B1, (**b**) HT339B1, (**c**) HT268B1, (**d**) HT265B1. The x- and y-axes represent spatial coordinates. The color bar indicates the inferred tumor proportions.

We evaluated CalicoST’s ability to infer CNAs and cancer clones in tumors with high normal cell admixture (low tumor cellularity) and complex spatial organization. Pancreatic tumors are notorious for having both features (for example, ref. ^36^), and two patients with pancreatic ductal adenocarcinoma (HT270P1 and HT288P1) had insufficient tumor purity in the bulk WES samples to identify CNAs. On the SRT data from these patients, CalicoST identified multiple cancer clones (Extended Data Fig. 1a,c) with LOH regions exhibiting clear differences in BAF (Extended Data Fig. 1b,d). For example, CalicoST identifies a deletion in chromosome 10 (chr10) unique to clone 2 in HT270P1, a chr4 deletion unique to clone 1 in HT288P1, and a chr8 deletion unique to clone 2 in HT288P1. Additionally, breast cancer sample HT339B1 (Extended Data Fig. 3) exhibits a complex geometry with diffuse cancer cells across the tissue slice, and CalicoST infers a tumor proportion for each spot that is consistent with the annotations of tumor/normal labels (AUC = 0.81; Supplementary Table 5). By explicitly modeling tumor proportions in its probabilistic model (7)(8), CalicoST achieves highly reliable CNAs in this sample: 88% genomic bins have identical allele-specific copy number from CalicoST analysis of SRT data and HATCHet2 analysis of WES data (Fig. 2 and Extended Data Fig. 2).

CalicoST identifies large-scale changes in tumor ploidy that are often challenging to infer, particularly for methods that infer only total copy numbers^15,17^. CalicoST identified near-triploid genomes in three patients with CRC liver metastasis (HT112C1, HT225C1 and HT230C1; Extended Data Fig. 2). For example, patient HT230C1 with CRC liver metastasis (Fig. 2c) has 39.4% and 15.1% of genomic bins with allele-specific copy number of {2, 1} and {2, 2} respectively (Fig. 2c), which agrees with the CNAs inferred from the matched WES sample (Supplementary Fig. 5). Previous studies have shown an association between triploidy and worse prognosis/poor survival^37^. Allelic information is key to the identification of near-triploid genomes from gene expression data; methods that only infer total changes in copy numbers miss many regions with a copy number of {2, 1} because the transcript counts in these regions may not differ substantially from copy number neutral regions, particularly because the gene expression signal is highly variable across the genome (Supplementary Fig. 1c).

CalicoST also revealed tumor heterogeneity and clone-specific copy number alterations that were missed in bulk WES data. On patient HT260C1 with CRC liver metastasis, CalicoST-identified CNAs were unique to cancer clones, for instance, a unique LOH of chr2 in clone 3, an LOH of chr8 in clones 2 and 3 but not in clone 1 (Fig. 2d). Both events are supported by BAF values (Supplementary Fig. 6 and Fig. 2e). The chromosome 8q region was assigned to a copy number of {3, 0} by HATCHet2 in the bulk WES (Fig. 2f), but its BAF and read depth ratio (RDR) measurements of chromosome 8q highlight an unusual deviation from the expected BAF value of {3, 0} copy number state (Fig. 2g), supporting CalicoST’s hypothesis that this region has undergone distinct allele-specific copy numbers in different cancer clones.

### CalicoST achieves high accuracy and spatial coherence

We compared CalicoST with existing methods for identifying CNAs from scRNA-seq data^15,28^ and spatial transcriptomics^17^, evaluating both the accuracy of inferred CNAs and the spatial distribution of inferred cancer clones. Specifically, we compared CalicoST with: Numbat^16^, an allele-specific CNA inference method for scRNA-seq data; STARCH^17^, a total copy number inference method for SRT data; and inferCNV^15^, a total copy number inference method for scRNA-seq data. Numbat and STARCH do not output integer copy numbers but rather copy number states (for example, amplification, deletion), which are not directly comparable with CalicoST and HATCHet2. Thus, to perform a comparison, we projected the integer allele-specific copy numbers from CalicoST and HATCHet2 to copy number states (Supplementary Text 12 and 16).

**Fig. 3:**
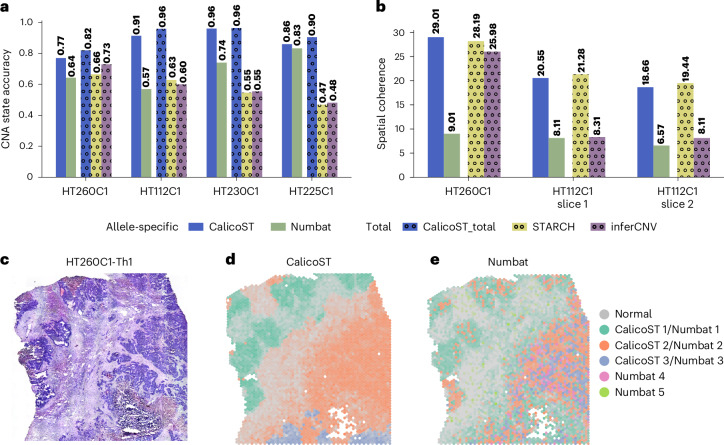
Accuracy and spatial coherence of inferred clones for CalicoST and other CNA inference methods. **a**,**b**, Accuracy (**a**) and spatial coherence (**b**) comparison among CalicoST, Numbat, InferCNV and STARCH on CRC liver metastasis patient samples. Solid bars indicate predictions of allele-specific copy number states, and dotted bars indicate predictions of total copy number states. **c**, H&E image of a CRC liver metastasis sample HT260C1. **d**, Cancer clones inferred by CalicoST. *x* and *y* axes are spatial coordinates, and the grayscale represents the proportion of normal cells within each spot, as inferred by RCTD. Other colors indicate cancer clones. **e**, Cancer clones inferred by Numbat using the same color scheme as in **d**.

**Extended Data Fig. 4:**
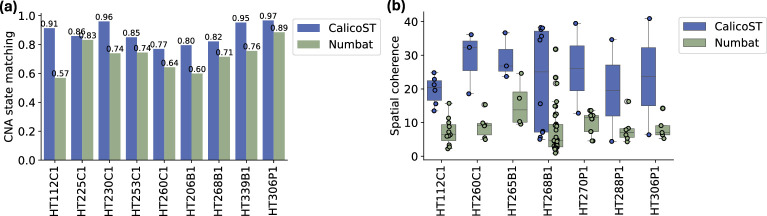
Accuracy and spatial coherence comparison between CalicoST and Num- bat on all HTAN patients. (**a**) Accuracy of the allele-specific copy number states inferred by CalicoST and Numbat on nine HTAN patients where ‘ground truth’ CNAs were inferred from matched WES data. (**b**) Spatial coherence of the cancer clones inferred by CalicoST and Numbat. The spatial coherence is evaluated by the z-score of joincount statistics, with higher values indicating a greater degree of spatial coherence. Each point represents a cancer clone within each slice of each patient (x-axis). As the two methods identify different numbers of clones, the two boxes include varying numbers of points for each patient. From left to right, the numbers of points in the boxplots are: HT112C1 (6 for CalicoST and 11 for Numbat), HT260C1 (3 for CalicoST and 6 for Numbat), HT265B1 (3 for CalicoST and 4 for Numbat), HT268B1 (10 for CalicoST and 39 for Numbat), HT270P1 (2 for CalicoST and 12 for Numbat), HT288P1 (2 for CalicoST and 6 for Numbat), HT306P1 (2 for CalicoST and 5 for Numbat). The upper and lower bounds of the box denote the 25% and 75% quantiles, the center line denotes the median, and the lower (upper) whiskers denote the smallest (largest) value within 1.5 times the IQR (interquartile range).

Focusing on four CRC liver metastasis samples with the most CNA events, CalicoST had the highest accuracy on all samples, outperforming Numbat in the inference of allele-specific copy numbers (average 25% higher accuracy) and outperforming STARCH (average 59% higher accuracy) and inferCNV (average 90% high accuracy) in inference of total copy numbers (Fig. 3a). We further compared the two allele-specific inference methods on all nine patients from HTAN: CalicoST was 23% more accurate than Numbat on average and had better accuracy for all nine patients (Extended Data Fig. 4).

The spatial distribution of cancer clones inferred by CalicoST was substantially more coherent than Numbat and inferCNV on the two CRC liver metastasis patient samples where all methods identify multiple cancer clones (Fig. 3b, Extended Data Fig. 4 and Supplementary Text 13). For example, on patient HT260C1 (Fig. 3c), CalicoST identified three spatially coherent clones: clone 1 on the top left, clone 2 on bottom right with normal spots (indicated in gray) separating these clones, and clone 3 in the bottom (Fig. 3d). In contrast, Numbat identified cancer clones with lower spatial coherence; for example, the orange cancer clone appears speckled throughout tumor regions on both the left and right of the normal spots that bisect the tissue slice (Fig. 3e).

### CalicoST infers tumor evolution in 3D space

We applied CalicoST to infer a phylogeographic tree in three-dimensional (3D) space (phylogeography in short) for two patients from HTAN with multiple adjacent slices of 10x Genomics Visium Spatial Transcriptomics data: patient HT112C1 CRC liver metastasis sample with two slices separated by 60 μm and patient HT268B1 breast cancer sample with five slices, with a distance of 100 μm between four of the slices, and an unknown distance between the first two slices. We aligned adjacent sections and derived a multi-slice alignment using PASTE2 (ref. ^38^), which was input into CalicoST.

**Fig. 4:**
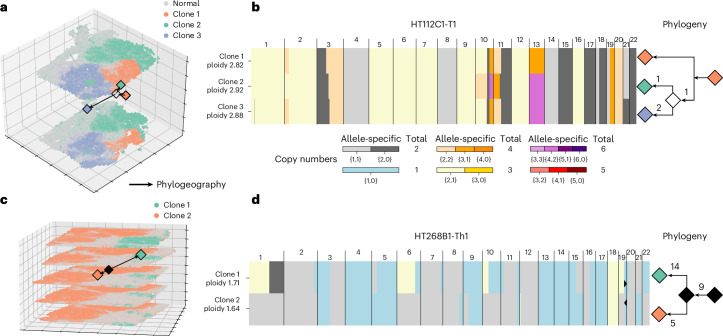
Tumor evolution in 3D inferred by CalicoST for patients HT112C1 and HT268B1. **a**, Spatial distribution and phylogeographic tree of three cancer clones inferred by CalicoST in two adjacent slices from patient HT112C1 with CRC liver metastasis. The grayscale indicates the inferred proportion of normal cells within each spot. Diamonds are the spatial centroid of each clone or inferred ancestor, and arrows indicate the inferred directions of tumor development. The distance between two slices in the *z*-coordinate is enlarged for clearer visualization. **b**, Allele-specific copy number profiles for the three cancer clones and the corresponding phylogeny (right) with branches in the phylogeny labeled by the number of unique large LOH events that occur on the branch. **c**, Spatial distribution and phylogeographic tree of two cancer clones inferred by CalicoST in five adjacent slices from patient HT268C1 with breast cancer. Color scheme is the same as **a**. **d**, Inferred allele-specific copy numbers and tumor phylogeny.

For the patient with CRC liver metastasis, CalicoST identified three spatially coherent clones in the 3D tumor tissue and inferred a phylogenetic tree from the CNAs in these clones (Fig. 4a). This phylogeographic tree shows the expansion of the tumor, branching from the ancestral clone 1 (orange) to two clones on either side (green and blue). The three clones have distinct allele-specific copy number profiles (Fig. 4b). Specifically, the blue clone 3 has a unique LOH on chr21, and the green clone 2 has a unique imbalanced CNA event on chromosome 11p. Both events are supported by a strong allelic imbalance in the BAF (Supplementary Fig. 7a). We observe a high consistency in clone composition and localization between the two slices, which is not surprising as the distance between the two slices (60 μm) is small and almost the same as the diameter of a spot within one slice (55 μm).

CalicoST identified two cancer clones in patient HT268B1 with breast cancer across five slices that are aligned in 3D space and reconstructs a phylogeography between the two clones (Fig. 4c). A mirrored deletion of chr19 distinguishes the clones (Fig. 4d) and is supported by the RDR and BAF values (Supplementary Fig. 7b). The phylogeography indicates that the ancestor (black diamond) is located between the two clones with a closer physical distance to clone 2 due to the fewer unique LOH events compared to clone 1. The spatial evolution of this tumor has a strong component in the *z*-axis direction, demonstrating the advantages of the 3D phylogeographic reconstruction.

We evaluated CalicoST’s accuracy on tumor slices with larger spatial separation by analyzing a subset of two slices (slices 1 and 4), with a separation of larger than 200 μm from patient HT268B1. The inferred cancer clones were largely concordant with those inferred from all five slices (adjusted Rand index = 0.996; Supplementary Fig. 8a). Additionally, allele-specific copy number profiles inferred by CalicoST on the two slices match the CNAs inferred from WES data for 79.2% and 68.0% of the genome, respectively (Supplementary Fig. 8b), with similar accuracy to that obtained from inference from all five slices. The BAF is a key signal to identify clones from distant slices: the BAF values in slice 1 are consistent with slice 4 for each of the clones (Supplementary Fig. 8c). This result demonstrates that CalicoST can be used to analyze 3D cancer clones from distant slices.

### Mirrored subclonal CNAs in a cancerous prostate organ

**Fig. 5:**
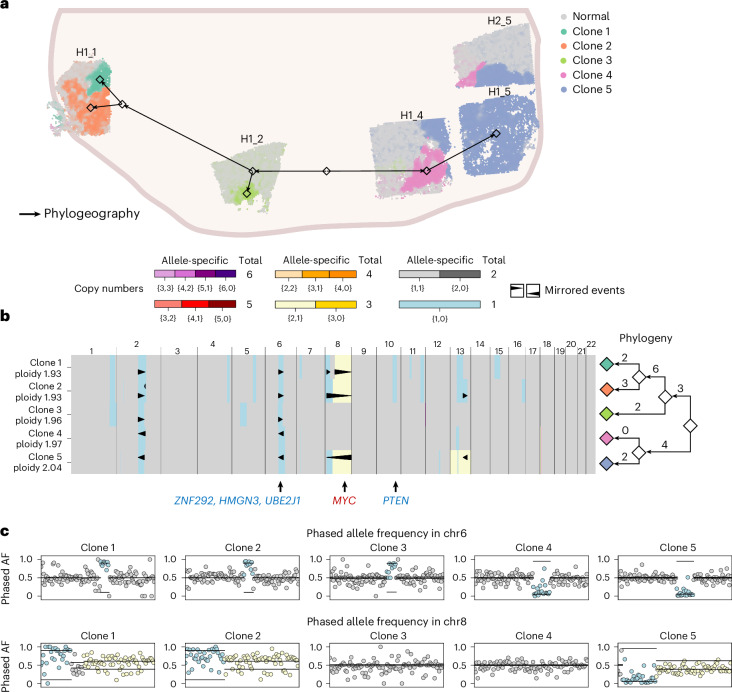
CalicoST infers a phylogeography and mirrored CNA events in a multi-section prostate cancer sample. **a**, Spatial distribution of cancer clones inferred jointly by CalicoST across five slices from a cancerous prostate. Positioning of five slices is according to ref. ^39^. Colors indicate inferred clones, including the normal clone in gray. Arrows represent the phylogeography of tumor evolution. **b**, Allele-specific copy number profiles for the five cancer clones and the corresponding phylogeny with branches in the phylogeny labeled by the number of unique large LOH events that occur on the branch. Colors indicate allele-specific copy numbers. The orientation and position of triangles indicate mirrored CNA events. **c**, BAF of each clone in chr6 and chr8. Colors indicate allele-specific copy numbers using the same color scheme as in **b**.

**Extended Data Fig. 5:**
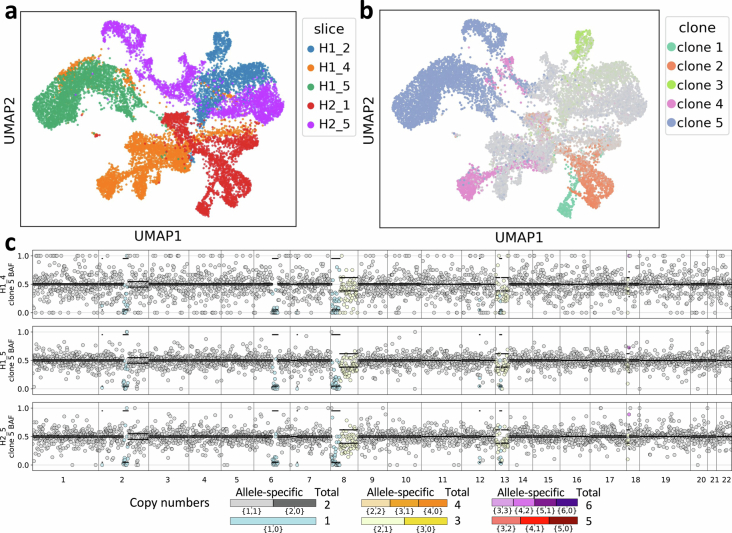
CalicoST identifies cancer clones across multiple slices of multi-section prostate cancer samples. (**a**) UMAP of gene expression in spots from five slices of a multi-section prostate cancer patient (without applying any batch effect correction or integration tools). Each point represents a spot, colored by the slice location. (**b**) UMAP of gene expression from five slices, with each spot (point) colored according to the clone assignment from CalicoST. Grayscale indicates the inferred tumor proportion, with more gray representing a higher proportion of normal cells. (**c**) BAF along the genome for spots assigned to clone 5 from three slices (H1 4, H1 5, and H2 5) from the right portion of the prostate. Each point represents a genomic bin, colored by the inferred allele-specific copy numbers from CalicoST.

We applied CalicoST to infer allele-specific CNAs and a phylogeography from five slices from a cross-section of a cancerous prostate^39^ (‘Running CalicoST on SRT data’). CalicoST identifies five cancer clones across the SRT slices (Fig. 5a) with distinct copy number profiles (Fig. 5b), each of which is supported by the BAF in each clone (Supplementary Fig. 9). The spatial distribution of the inferred cancer clones is visually consistent with the pathologist-annotated tumor regions shown in ref. ^39^. Notably, clone 5 (Fig. 5a) is shared across all three slices (H1_4, H1_5 and H2_5) on the right side of the prostate, and forms a contiguous spatial region, even though CalicoST was not provided with information about the relative locations of slices in the prostate. The consistent BAF values in the spots assigned to clone 5 from different slices supported the grouping of these spots into the same clone (Extended Data Fig. 5c). This shared clone across multiple slices is difficult to detect from clustering of gene expression across spots, where clone 5 spots split into multiple clusters (Extended Data Fig. 5a,b), possibly due to batch effects or biological differences between slices. This demonstrates the advantage of CalicoST’s joint inference across multiple slices, and the robustness of the BAF signal across slices.

The clones on the two halves of the prostate cross-section (left and right) are distinguished by multiple CNAs. Most prominent among these are four mirrored CNA events, on chromosomes 2, 6 and 8 where clones 1 and 2 have different amplified/deleted alleles than clones 4 and 5 (Fig. 5c). Specifically, chromosome 8p, one of the most frequently deleted regions in prostate cancer^40^, has a mirrored deletion, and chromosome 8q has a mirrored amplification containing the *MYC* gene, a well-known oncogene in aggressive prostate cancer. Also, chr6 has a mirrored deletion of a commonly deleted region in prostate cancer^41^, harboring *ZNF292*, *HMGN3* and *UBE2J1*, three reported tumor suppressor genes^42^. Mirrored subclonal CNAs suggest possible convergent evolution with independent deletions in different tumor clones^26^. In contrast, the original published analysis of this data^39^ used SpatialInferCNV—which is based on InferCNV^15^ and identifies only total copy numbers—and concluded that the deletion in chromosome 6q is a truncal event, missing the differential loss of the two alleles in the two halves of the prostate.

**Extended Data Fig. 6:**
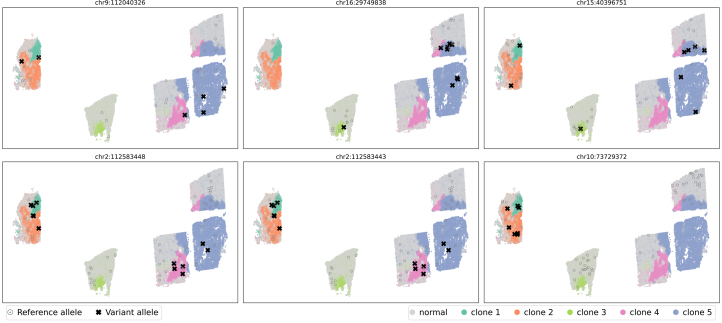
Spatial localization of six identified somatic SNVs in the multi-sample prostate cancer patient. Each spot is colored by the clone inferred by CalicoST, with gray indicating normal spots. Spots containing the variant allele of the somatic SNV are marked by a black cross. Spots containing the reference allele are marked by a gray circle. The first five SNVs are inferred to be truncal SNVs present in both the left and right sides of the prostate, while the sixth SNV (bottom right) is inferred to be present in only the left side.

The inferred phylogeography splits the cancer clones into two main lineages, which coincide with the left and right partitioning of the slices in physical space (Fig. 5a). On the left half, clone 3 contains fewer CNA events and is closer to a normal diploid genome than clones 1 and 2, while clones 1 and 2 share many deletions in chromosomes 1, 2, 4, 5, 6, 8, 10 and 11. On the right half, clone 4 shares multiple CNAs with clone 5, but does not have any unique CNAs and thus clone 4 is marked as an ancestor of clone 5, which is consistent with its spatial location closest to the root. Interestingly, the absence of truncal CNA events and the clear bifurcation in both genetic and physical space suggests that the tumor on the left and right halves diverged in a very early stage and had relatively independent evolution. Consistent with this hypothesis, we identified six high-confidence somatic SNVs across the slices (‘Identifying somatic SNVs in multi-section prostate cancer’). Five SNVs are shared between the left side and right side of the prostate (Extended Data Fig. 6), and are likely truncal events that are shared by all cancerous cells in the tumor. One SNV is unique to clones 1 and 2 on the left half of the prostate (Extended Data Fig. 6; chr10:73729372), supporting the phylogeny inferred using CalicoST CNAs, where clones 1 and 2 are two sibling nodes.

### CalicoST analysis of Slide-tags data and simulated data

**Extended Data Fig. 7:**
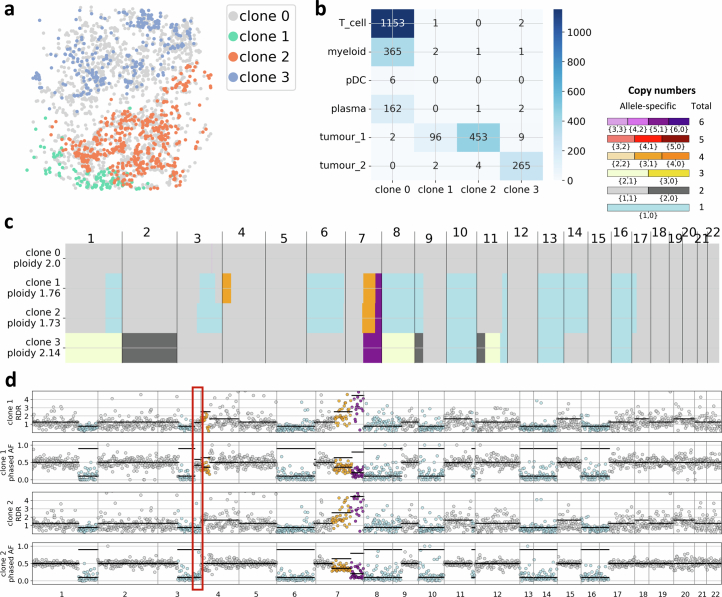
CalicoST identifies cancer clones and allele-specific CNAs from Slide-tags data of a human melanoma sample. (**a**) Spatial organization of normal (clone 0) and three tumor clones (clones 1-3) inferred by CalicoST on a human melanoma sample sequenced using Slide-tags. (**b**) Compar- ison of cell type labels for each location from^43^ and clone labels inferred by CalicoST. (**c**) Allele-specific copy numbers inferred by CalicoST for each clone. (**d**) RDR and BAF along the genome for clones 1 and 2. Colors indicate the allele-specific copy number of the corresponding genomic bin. Red box highlights a LOH event on chr3q that is unique to clone 2.

We used CalicoST to analyze a human melanoma sample assayed using the Slide-tags technology^43^, which achieves single-cell spatial resolution and can perform spatial multiomics using the 10x Genomics Multiome ATAC + Gene Expression platform. Using the RNA data, CalicoST identified three spatially coherent cancer clones (Extended Data Fig. 7 and Supplementary Text 14), with one additional clone that was not reported in the original study^43^.

We evaluated the accuracy of CalicoST using two sets of simulated data. First, we generated 90 simulated samples with varying numbers and lengths of CNAs within each cancer clone, and observed that CalicoST achieved the highest accuracy in both identifying cancer clones and identifying CNAs compared to Numbat and STARCH (Supplementary Fig. 11 and Supplementary Text 15). Next, we generated 135 simulations with varying complexity of spatial organization and found that CalicoST achieved a high accuracy in distinguishing between cancer and normal cells (Supplementary Fig. 12).

## Discussion

We introduced CalicoST, an algorithm that infers allele-specific copy numbers and reconstructs a phylogeography relating cancer clones in time and space using SRT data. CalicoST uses inferred LOH events to construct a phylogeny relating the cancer clones and a phylogeography in space, integrating both genomic and spatial evolution of a tumor in a unified model. When multiple slices are available, CalicoST infers a joint phylogeography across the slices including 3D models of spatial tumor evolution.

CalicoST has several limitations and directions for future improvement. First, CalicoST requires sufficient coverage of heterozygous SNPs to obtain informative BAF values. Thus, lower-coverage SRT technologies such as Slide-seqV2, which has only ≈30 SNP-covering reads per spot in published datasets^22^, require aggregating counts from neighboring spots with a corresponding loss of spatial resolution before applying CalicoST. Moreover, probe-based technologies such as 10x Genomics Visium CytAssist for analysis of formalin-fixed paraffin-embedded tissues do not sequence SNPs and thus allele-specific CNAs cannot be identified. Second, the length of the CNAs that are reliably detected is limited by the sequencing coverage as well as the inherent difficulties in detecting DNA aberrations from gene expression data. This resolution depends on the gene density within a genomic region as well as the number of spots that contain the aberration. However, it is nearly impossible to detect aberrations that contain only a single gene, as these are indistinguishable from differential expression. Third, CalicoST’s use of LOH events as phylogenetic markers helps infer accurate phylogenies, but requires that a tumor sample have enough of these events. Leveraging other CNAs in phylogeny reconstruction may allow phylogeny inference on additional tumor samples, but requires accurate models of copy number evolution. Fourth, CalicoST currently struggles with inferring accurate integer copy numbers for high-copy amplifications due to the high variance in gene expression. For example, CalicoST infers chr13 of HTAN patient HT260C1 to have three and four total copies across the inferred cancer clones but the total copy numbers inferred from WES data by HATCHet2 is five copies. Fifth, the runtime of CalicoST could be further improved with faster optimization algorithms or GPUs; current runtime is between 2 and 8 hours (Supplementary Fig. 13) with the bottleneck being the parameter fitting to the negative binomial and beta-binomial distribution. Sixth, further improvements can be made in the model selection criteria for selecting the number of clones (Supplementary Text 10) and the spatial coherence parameter in the HMRF (Supplementary Text 5). Particularly, for tumor samples containing cancerous cells with little spatial organization, a strong spatial coherence prior may lead to inaccurate inference of CNAs and cancer clones. Finally, CalicoST could be extended to other spatial measurements, such as spatial ATAC-seq, which combines the assay for transposase-accessible chromatin and sequencing, or to incorporate additional modalities such as matched WES data or lineage-tracing experiments^44^ combining the advantages and overcoming the limitations of different technologies.

The use of SRT in cancer analysis is growing rapidly. CalicoST can help bring valuable insights into copy number drivers of cancer, spatial tumor heterogeneity and spatial evolution, serving as a foundation for additional biological analyses integrating genetic evolution, epigenetic changes and spatial organization of cell types. CalicoST could also be used to study drug resistance from new angles by characterizing differences in spatial structure and microenvironment between drug-resistant and drug-sensitive clones.

## Methods

### CalicoST workflow

CalicoST has the following main steps (Supplementary Fig. 14). In a preliminary step (step 0), CalicoST uses a panel of known germline heterozygous SNP loci (currently obtained from the 1000 Genomes Phase 3) and extracts the counts for reference and alternative alleles, which provides key information for inferring allele-specific CNAs. CalicoST infers an initial haplotype (also known as phasing) of each SNP using a database of reference haplotypes and a population-based SNP phasing algorithm, Eagle2 (ref. ^45^). The resulting phasing is generally of high quality for nearby SNPs but prone to switch errors for distant SNPs (Supplementary Text 1). In step 1, CalicoST next attempts to correct potential errors in the population-based phasing using the BAF computed by combining allele counts from all spots; that is, creating a pseudobulk sample (‘Phasing and binning’), because all cells have identical maternal/paternal haplotypes. Then, CalicoST partitions the genome into nonoverlapping consecutive bins and aggregates the transcript counts and allele counts according to the phasing of SNPs within each bin. In step 2, CalicoST identifies normal spots either directly from an inferred tumor purity per spot (‘Inferring tumor proportions using BAF’) or by an initial clustering of spots by the phased BAF and identifying the cluster whose center is closest to BAF = 0.5 (Supplementary Text 7). The inferred normal spots provide baseline gene expression in normal cells; higher-than-baseline expression is potentially due to copy number gains and lower-than-baseline expression indicates copy number losses. Additionally, we use normal spots to remove genomic bins that potentially have allele-specific gene expression irrelevant to CNAs (Supplementary Text 8). In step 3, CalicoST clusters genomic bins into ‘copy number states’ and infers cancer clones simultaneously (‘CNAs and clone inference problem’; Supplementary Text 2–6). CalicoST explicitly models the correlation among genomic bins along the genome using the HMM and among the cancer clones in space using the HMRF. Briefly, CalicoST estimates a latent value for the RDR—corresponding to the relative copy numbers compared to diploid—and a latent value for the BAF—indicating the imbalance of copy numbers between the two alleles—for each hidden copy number state in the HMM. CalicoST heuristically selects the number of cancer clones by identifying and merging clones with similar copy number profiles using the latent RDR and BAF values inferred using a user-defined number of initial clones (Supplementary Text 10). In step 4, CalicoST finds allele-specific integer copy numbers for each copy number state that best explain the inferred latent RDR and BAF (Supplementary Text 9). In the final step 5, CalicoST reconstructs a tumor phylogeography by inferring a phylogeny using the inferred LOH events and projecting to space (‘Reconstructing tumor phylogeography’).

We provide further details for step 1 (‘Phasing and binning’), step 3 (‘CNAs and clone inference problem’) and step 5 (‘Reconstructing tumor phylogeography’) of CalicoST below. See Supplementary Text for details of other steps.

In the case when CalicoST is asked to estimate tumor purity for each spot, CalicoST first identifies genomic regions that have an LOH by running step 1 through step 3 assuming each spot contains homogeneous normal cells or clones, and then estimates tumor purity using the BAF (‘Inferring tumor proportions using BAF’). The details of analyses on HTAN (WashU cohort), multi-section prostate cancer samples and the Slide-tags sample are described in ‘Running CalicoST on SRT data’ and ‘Identifying somatic SNVs in multi-section prostate cancer’.

### Phasing and binning

The preliminary step (step 0) derives a population-phased allele count matrix **Y**^0^ of haplotype 1 and a total allele count matrix **D**^0^ containing the sum of counts of two haplotypes; each row indicates a genomic locus and each column indicates a spot. When computing these matrices, CalicoST excludes spots where the total unique molecular identifier (UMI) counts or SNP-covering UMI counts are too low based on a user-specified threshold, which was set to 50. CalicoST excludes the following genes: (1) lowly expressed genes that are expressed in less than a user-provided percentage of spots (we used 0.5% for all datasets analyzed in the paper), (2) genes with too high expression detected by the outlier detection algorithm, local outlier factor^46^, (3) immunoglobulin genes (212 genes) and/or genes within HLA regions (21 genes), which are known to have large variability in expression even without CNAs. CalicoST further excludes SNPs that are located in intergenic and HLA regions.

Haplotypes inferred by population-based phasing are prone to switch errors for distant SNPs, and thus in step 1, CalicoST infers a new haplotype label for each SNP based on **Y**^0^ and **D**^0^ (Supplementary Fig. 15a–c). Because all spots have identical maternal/paternal haplotypes, CalicoST computes BAF values by combining counts from all spots (that is, creating a pseudobulk sample) and obtaining ${{\bf{y}}}^{\,0}={\sum }_{s}{Y}_{g,s}^{0}$ and ${{\bf{d}}}^{0}={\sum }_{s}{D}_{g,s}^{0}$ across spots *s* to reduce the sparsity of allele counts (see Supplementary Fig. 15d as an example of HTAN patient HT260C1). Large CNAs that lead to imbalanced copy number states result in continuous blocks of SNPs with BAF values ≠ 0.5. Assuming population-based phasing is generally accurate for nearby SNPs, CalicoST models the observed BAF values in the pseudobulk sample as the observations of the HMM whose hidden states correspond to mirrored allele-specific copy number states as follows.

Let **h** = [*h*_*g*_] ∈ {haplotype 1, haplotype 2}^*G*^ denote the phasing of each SNP loci *g*. We define the hidden states of the HMM model as a pair of phasing *h*_*g*_ and latent BAF cluster *z*_*g*_. We model the observed B allele count at the *g*^th^ SNP loci, ${{\bf{y}}}_{g}^{0}$, by

$$\begin{array}{c}{{\bf{y}}}_{g}^{0}| {h}_{g}=\,\text{haplotype 1}\,;{z}_{g}=i \sim{\mathrm{BetaBinom}}({{\bf{d}}}_{g}^{0},\tau {p}_{i},\tau (1-{p}_{i}))\\ {{\bf{y}}}_{g}^{0}| {h}_{g}=\,\text{haplotype 2}\,;{z}_{g}=i \sim{\mathrm{BetaBinom}}({{\bf{d}}}_{g}^{0},\tau (1-{p}_{i}),\tau {p}_{i}),\end{array}$$

which give the emission probabilities of the HMM. We assume the elements *h*_*g*_ and *z*_*g*_ of the hidden state pair are independent in the start probability and state-transition probability:

$${\mathbb{P}}(({z}_{g},{h}_{g})| ({z}_{g-1},{h}_{g-1}))={\mathbb{P}}({z}_{g}| {z}_{g-1}){\mathbb{P}}({h}_{g}| {h}_{g-1}).$$

The transition among BAF states and phase is defined, respectively, as follows:

$$\begin{array}{c}{\mathbb{P}}({z}_{g}| {z}_{g-1})=\left\{\begin{array}{ll}t\quad &({z}_{g}={z}_{g-1})\\ \frac{1-t}{K-1}\quad &({z}_{g}\ne {z}_{g-1}).\end{array}\right.\\ {\mathbb{P}}({h}_{g}| {h}_{g-1})=\left\{\begin{array}{ll}q\quad &({h}_{g}={h}_{g-1})\\ 1-q\quad &({h}_{g}\ne {h}_{g-1}).\end{array}\right.\end{array}$$

We estimate the parameters and perform MAP estimation of the hidden states using the Baum–Welch algorithm. Using the pseudobulk sample, the HMM outputs one phase vector **h** across all spots. In contrast, Numbat^28^ infers an independent phase along with the CNA states for each cancer clone, potentially inducing errors in both phasing and CNA inference.

Using the phasing estimated by the HMM model, CalicoST next aggregates counts for each haplotype across SNPs in genomic bins. Specifically, CalicoST partitions the genome into consecutive nonoverlapping bins and computes a phased allele count *Y*_*j*,*s*_ of haplotype 1 for each genomic bin *j* and spot *s* and a total count *D*_*j*,*s*_ by aggregating counts from *Y*^0^ and *D*^0^ as follows.

$$\begin{array}{c}{D}_{j,s}=\sum\limits_{g\in J}{D}_{g,s}^{0}\\ {Y}_{j,s}=\sum\limits_{g\in J}\left({\mathbb{1}}[{h}_{g}=\,\text{haplotype 1}\,]{Y}_{g,s}^{\,0}+{\mathbb{1}}[{h}_{g}=\,\text{haplotype 2}\,]({D}_{g,s}^{0}-{Y}_{g,s}^{\,0})\right)\end{array}$$

Additionally, CalicoST aggregates counts **X**^0^ of transcripts that located within each genomic bin to **X**. Applying the variable-length binning introduced by ref. ^34^, we determine the size of each bin by requiring that the sum of total allele counts within each bin across all spots exceeds a user-defined minimum, except when the bin is located at the end of chromosomes. This binning procedure generates sufficient counts to infer CNAs in later steps of CalicoST (Supplementary Fig. 15f). Phasing and binning allow distinguishing small changes in the phased BAF values. For example, on HTAN patient HT260C1, a small deviation in the BAF from the balanced value 0.5 in clone 2 chr3 is clearly distinguished from a large deviation in chr4 and chr5 and from no deviation in chr11 (Supplementary Fig. 15g). We call the inferred haplotype 1 as B haplotype/allele, and haplotype 2 as A haplotype/allele. Correspondingly, the copy numbers of the two haplotypes are the B copy and the A copy.

### CNAs and clone inference problem

Given the spatial coordinates ${\bf{S}}\in {{\mathbb{R}}}^{N\times 2}$, aggregated transcript count matrix **X** = [*x*_*g*,*n*_], phased B allele count matrix **Y** = [*y*_*g*,*n*_] and total allele count matrix **D** = [*d*_*g*,*n*_] across *n* = 1, …, *N* spots and *g* = 1, …, *G* genomic bin, CalicoST finds a clone label vector ***ℓ*** ∈ {1, …, *M*}^*N*^ to indicate the corresponding copy number profile (clone) for each spot, and two allele-specific copy number matrices for each clone for each bin, **A** = [*a*_*g*,*m*_] of A allele copies, and **B** = [*b*_*g*,*m*_] of B allele copies. If multiple SRT slices are available, **S**, **X**, **Y** and **D** are the concatenated matrices across the slices.

CalicoST solves the problem of inferring maximum likelihood estimates of ***ℓ***, **A** and **B**. In addition to SRT data, the optional inputs to this problem are alignment *W* of multiple slices and the ‘tumor proportions’ ***θ*** = [*θ*_*n*_] ∈ [0, 1]^*N*^ for each spot. CalicoST solves the CNA and clone inference problem.

**CNA and clone inference problem.** Given SRT data (**X**, **Y**, **D**, **S**), an alignment *W* of multiple slices (optional), tumor proportions ***θ*** (optional) and the number *M* of clones, find clone labels ***ℓ*** and integer allele-specific copy numbers **A** and **B** that maximize the log-likelihood of the data as shown in equation (1):

1 $$\mathop{{\mathrm{arg}}\,{\mathrm{max}}}\limits_{\begin{array}{c}{\boldsymbol{\ell }}\in {\{1,\ldots ,M\,\}}^{N}\\ {\bf{A}},{\bf{B}}\in {{\mathbb{Z}}}^{G\times M}\end{array}}{\mathbb{P}}({\bf{X}},{\bf{Y}}| {\boldsymbol{\ell }};{\bf{A}},{\bf{B}},{\boldsymbol{\lambda }},{\bf{D}},({\boldsymbol{\theta }}),(W)){\mathbb{P}}({\boldsymbol{\ell }};{\bf{S}}).$$

In the notation of probabilistic models, we separate the conditional random variables from the parameters and constants by a semicolon, that is, ***ℓ*** and **Z** are random variables in equation (1), and **A**, **B**, ***λ***, **D**, ***θ*** and *W* are parameters or constants. We also put the optional inputs in parentheses.

Solving this problem with integer-valued **A** and **B** is challenging. Notably, the probabilistic model for **X** and **Y** involves fractions derived from **A** and **B** (Supplementary Text 2). Earlier works on copy number inference^4,29,34,47,48^ transform integer copy numbers into a discrete set of real-valued latent parameters: read depth ratio (RDR) ***μ*** and B allele frequency (BAF) **p**. We use the same parameter transformation and split the problem into two steps: CalicoST infers clone labels and the latent RDR and BAF parameters and CalicoST infers **A**, **B** from the estimated RDR and BAF parameters.

Allele-specific copy numbers only take values from a finite set of size *K*, which we call the ‘copy number states’. Accordingly, the latent RDR and BAF also have *K* unique values. We introduce a categorical variable **Z** = [*z*_*g*,*m*_] ∈ {1, …, *K*}^*G*×*M*^ to indicate which of the *K* copy number states each genome bin in each clone takes. CalicoST infers *K* RDR parameters ${\boldsymbol{\mu }}=[{\mu }_{k}]\in {{\mathbb{R}}}^{K}$, *K* BAF parameters ${\bf{p}}=[{p}_{k}]\in {{\mathbb{R}}}^{K}$, state indicator **Z** and clone labels ***ℓ*** solving the copy number state and clone inference problem.

**Copy number state and clone inference problem.** Given SRT data (**S**, **X**, **Y**, **D**), an alignment *W* of multiple slices (optional), tumor proportions ***θ*** (optional) and the number *M* of clones, find clone labels ***ℓ***, copy number states **Z**, latent RDR ***μ*** and BAF **p** that maximize the log-likelihood of the data as shown in equation (2):

2 $$\mathop{{\mathrm{arg}}\,{\mathrm{max}}}\limits_{\begin{array}{c}{\boldsymbol{\ell }}\in {\{1,\ldots ,M\,\}}^{N}\\ {\boldsymbol{\mu }},{\bf{p}}\in {{\mathbb{R}}}^{K}\\ {\bf{Z}}\in {\{1,\ldots ,K\}}^{G\times M}\end{array}}{\mathbb{P}}({\bf{X}},{\bf{Y}}| {\boldsymbol{\ell }},{\bf{Z}};{\boldsymbol{\mu }},{\bf{p}},{\boldsymbol{\lambda }},{\bf{D}},({\boldsymbol{\theta }}),(W)){\mathbb{P}}({\bf{Z}}){\mathbb{P}}({\boldsymbol{\ell }};{\bf{S}}).$$

We derived ${\mathbb{P}}({\bf{X}},{\bf{Y}}| {\boldsymbol{\ell }},{\bf{Z}};{\boldsymbol{\mu }},{\bf{p}},{\boldsymbol{\lambda }},{\bf{D}},({\boldsymbol{\theta }}),(W))$ under two different assumptions: when each spot contains a single clone (Supplementary Text 2) and when a spot contains a mixture of normal cells and cancer cells (Supplementary Text 3). We model the correlation of copy number states along the genome by specifying ${\mathbb{P}}({\bf{Z}})$ as a Markov chain (Supplementary Text 4), and the correlation of cancer clones in space by specifying ${\mathbb{P}}({\boldsymbol{\ell }})$ as a Markov random field (Supplementary Text 5). Overall, the likelihood as shown in equation (2) combines the HMM of copy number states with the HMRF of cancer clones.

CalicoST uses a block coordinate ascent method to optimize (equation (2)) and iteratively solve for ***ℓ*** and for ***μ***, **p**, **Z**. Given ***ℓ***, we solve for ***μ***, **p**, **Z** using the HMM in Supplementary Text 4; given ***μ***, **p**, **Z**, we solve for ***ℓ*** under the HMRF in Supplementary Text 5.

### Inferring tumor proportions using BAF

CalicoST includes an optional procedure to estimate the proportion of cancer cells at each spot from the BAF at genomic regions that are affected by LOH events. While many methods deconvolve cell-type proportions using a reference of cell-type-specific gene expression^35,49–51^, CalicoST leverages BAF values and achieves reference-free deconvolution of tumor and normal proportions. First, assume the genomic bins affected by LOH events are denoted by *R*, and further without loss of generality, assume that the B allele is lost. If a spot contains only cancer cells with the LOH, then BAF will be 0 for all genomic bins in the LOH region. If a spot contains only normal cells—which by definition do not have somatic CNAs—the BAF will be close to 0.5 on autosomes. Let *f* be the phased BAF at the LOH region of a given spot containing a mixture of cancer and normal cells, *f* will be between 0 and 0.5 and will the related to the tumor proportion *θ* and RDR *μ* according to equation (3):

3 $$f=\frac{0.5(1-\theta )}{\theta \mu +(1-\theta )}.$$

Equation (3) gives the following estimate of tumor proportion *θ* at a given spot using the observed BAF at LOH: $\theta =\frac{0.5-f}{0.5+\mu f-f}$. We apply this equation using a robust estimate of the BAF *f* across all LOH regions (possibly including multiple chromosomes), by combining the UMI counts from all genomic bins with LOH: $f=\frac{{\sum }_{g\in R}{Y}_{g,i}}{{\sum }_{g\in R}{D}_{g,i}}$.

In reality, the set of genomic bins affected by LOH is unknown a priori. We predict genomic bins that are likely to be affected by LOH in the tumor by running step 1 through step 3 of CalicoST (Supplementary Fig. 14) assuming that each spot contains a single clone (Supplementary Text 2) with only BAF values. This results in a clustering of genomic bins according to the estimated BAF values. We infer LOH regions as the clusters for which the estimated BAF differs from the value of 0.5 expected for balanced regions by a user-defined threshold (default value = 0.2).

### Reconstructing tumor phylogeography

CalicoST reconstructs a tumor phylogeography from the inferred allele-specific copy number numbers *A*, *B* in two steps. First, CalicoST builds a phylogeny between the cancer clones using the LOH events as phylogenetic markers. LOH events are irreversible phylogenetic characters: if an ancestor cancer cell loses a region of one allele, its descendant cells cannot regain that region. Additionally, LOH events are generally inferred with higher accuracy than other CNAs because these events have the most imbalanced BAF values (see Supplementary Table 4 for BAF values of LOH event in clone 1 of the multi-section prostate cancer sample). We construct a phylogenetic tree for highly confident LOH events under the irreversibility assumption using Startle^33^. In this construction, we restrict to LOH events that contain a user-defined minimum number of genomic bins (default value = 3) and are supported by a user-defined minimum number of SNP-covering UMIs across all spots (default value = 100) in the corresponding clone.

To infer a phylogeography, CalicoST projects the leaf nodes (which correspond to inferred clones) to the center of involved spots in space (denoted by *s*_*v*_ for node *v*), and infer the spatial location of ancestor nodes using a Gaussian diffusion model. Specifically, we assume the spatial distance between a node *v* and its parent *p*(*v*) in the phylogenetic tree follows a Gaussian distribution with a variance proportional to the number of mutations *w*_*v*,*p*(*v*)_ on the edge as shown in equation (4):

4 $${s}_{v} \sim {\mathcal{N}}({s}_{p(v)},{w}_{v,p(v)}I\,).$$

We estimate the ancestor locations in the phylogenetic tree by maximizing the joint probability of spatial locations of all nodes, ${\{{s}_{v}\}}_{v}$, under the above Gaussian distribution as shown in equation (5):

5 $$\arg \max \log {\mathbb{P}}({\{{s}_{v}\}}_{v})=\arg \max \sum _{v}\log {\mathbb{P}}({s}_{v}| {s}_{p(v)}).$$

Finally, we draw directed edges among the clone centers and inferred locations of ancestors according to the phylogenetic tree, where each edge is directed from the parent to the child.

### Running CalicoST on SRT data

We processed the SRT data from HTAN samples and the multi-section prostate cancer data using Space Ranger versions 1.1.0 and 2.0.0, respectively, to obtain the transcript count matrix and read alignment BAM files. For the Slide-tags data, we obtained the transcript count matrix and BAM file from ref. ^43^. The 12 HTAN patients, the patient with multi-section prostate cancer and the Slide-tags sample have varying numbers of spots and sequencing depths. Four patients (HT268B1, HT206B1, HT225C1 and the patient with multi-section prostate cancer) have Visium data from four or more slices and over 15,000 spots in total; the remaining patients only have one or two slices and less than 7,500 spots (Supplementary Table 2). The median UMI counts per spot range from 3289.5 (multi-section prostate cancer sample) to 28476.5 (sample HT112C1; Supplementary Fig. 16a). These patients also have varying numbers of SNP-covering UMIs per spot ranging from a median of 223.0 (multi-section prostate cancer sample) to 1786.5 (sample HT112C1; Supplementary Fig. 16b). CalicoST excludes spots that have UMI counts and SNP-covering UMI counts less than a user-defined threshold; we set a threshold of 50 for all datasets, leading to the post-quality-control summary statistics in Supplementary Table 2 and Supplementary Fig. 16. After the phasing and binning step of CalicoST, there are 1,200–2,700 bins along the genome (Supplementary Table 3). Each bin contains a median of 7–13 SNPs and 4–11 genes for Visium samples, and a median of 60 SNPs and 8 genes for the Slide-tags sample.

For HTAN patients with multiple slices obtained from a 3D tissue sample, we aligned the consecutive slices from the HTAN data using PASTE2 (refs. ^38,52^) and provided the alignment matrix *W* to CalicoST to infer CNAs and tumor clones in 3D space, as described in Supplementary Text 6. PASTE2 handles batch effects between slices by using both expression information and spatial information when performing the alignment. For the multi-section prostate cancer sample, where the slices are obtained from a large two-dimensional cross-section, we used CalicoST to jointly infer CNAs and cancer clones without providing alignment matrices. Because the Slide-tags sample had a high degree of normal cell infiltration, there was lower spatial coherence between cancer clones. To address this issue, we used a weighted adjacency matrix *W* in the HMRF setting *W* to be a weighted sum between the *k*-nearest-neighbor graphs of spots in spatial coordinates and expression space.

### Identifying somatic SNVs in multi-section prostate cancer

We identified confident somatic SNVs in the multi-section prostate cancer data as follows. First, we used cellsnp-lite^53^ to identify and count reads with non-reference nucleotides (in GRCh38) for each spot. Specifically, we followed the procedure in the cellsnp-lite tutorial to perform de novo variant calling on the pseudobulk of all spots followed by counting reads at the identified variant positions for individual spots. Second, we removed known germline SNPs in the 1000 Genomes Project phase 3 data using Bcftools^54^. Third, we removed putative SNVs in intergenic regions; 10x Visium data capture either the 3′ end or the 5′ end of RNAs, and thus the intergenic reads are likely to have sequencing or alignment errors. We also excluded candidate SNVs that had some evidence of being germline SNVs using the total and variant read counts in normal spots. Specifically, we marked a spot as normal if the tumor proportion estimated by CalicoST was below 0.5, and marked a candidate SNV as germline if it has a nonzero variant allele count in any normal spots, or if the total read count in normal spots is smaller than a threshold (we used 15). In the latter case, a low total read count is insufficient to rule out the possibility that the SNV is germline. Finally, we defined somatic SNVs as the remaining candidates with variant allele count ≥ 5 and tumor variant allele frequency > 0.3.

### Reporting summary

Further information on research design is available in the Nature Portfolio Reporting Summary linked to this article.

## Online content

Any methods, additional references, Nature Portfolio reporting summaries, source data, extended data, supplementary information, acknowledgements, peer review information; details of author contributions and competing interests; and statements of data and code availability are available at 10.1038/s41592-024-02438-9.

## Supplementary information

Supplementary Information Supplementary Text 1–18, Figs. 1–15 and Tables 1–5.

Reporting Summary

## Data Availability

Sequencing data are part of the HTAN dbGaP under accession phs002371.v3.p1. These data are available through the HTAN DCC Portal (https://data.humantumoratlas.org/) under the HTAN WUSTL Atlas. Sequencing data of the multi-section prostate cancer samples were obtained from the European Genome-phenome Archive under accession EGAS00001006124. The output data of CalicoST of the HTAN and multi-section prostate cancer data are available on Zenodo via 10.5281/zenodo.13667151 (ref. ^55^).

## Code availability

The code is publicly available at https://github.com/raphael-group/CalicoST/ under a BSD 3-Clause license. The version of code used in the analysis is available on Zenodo via 10.5281/zenodo.10986535 (ref. ^56^).
