# Supplementary material for: Inferring allele-specific copy number aberrations and tumor phylogeography from spatially resolved transcriptomics
Source: Nat Methods. 2024 Oct 30;21(12):2239–47. doi: 10.1038/s41592-024-02438-9 (PMC11621028; doi:10.1038/s41592-024-02438-9)
Supplement: Supplementary file 1 — Supplementary Text 1–18, Figs. 1–15 and Tables 1–5. [file 41592_2024_2438_MOESM1_ESM.pdf]

nature methods

Supplementary information

https://doi.org/10.1038/s41592-024-02438-9

Inferring allele-specific copy number aberrations and tumor phylogeography from spatially resolved transcriptomics

In the format provided by the authors and unedited

Supplementary Text

S1 Extracting allele and total counts

We applied the pipeline from Numbat [1] to extract allele counts from SRT data ignoring spatial information. The first step is to count the number of UMIs covering candidate heterozygous SNPs for each SNP within each spot using cellsnp-lite [2], where the candidate heterozygous SNPs are given by 1000 Genomes Project phase 3 data [3]. This step is also known as "genotyping". If there are multiple slices from a patient, we jointly genotype across all slices by running cellsnp-lite on a concatenated BAM file from all slices.

The second step is to categorize the candidate SNPs into three categories (homozygous, heterozygous, and unsure) using a pseudobulk of all spots, as not all SNPs in the 1000 Genomes Project are heterozygous in the SRT sample. We assume the SNP is very likely to be heterozygous if we observe sufficient UMIs for both alleles, and label it as heterozygous if there are at least 2 UMIs for each allele across all spots and the frequency of alternative alleles deviates from both 0 and 1 by a threshold (which is 0.1 by default). We assume a SNP is homozygous if it only has one observed allele containing sufficient SNP-covering UMIs (10 UMIs). The remaining SNPs are categorized as unsure.

The third step is to preliminarily phase the heterozygous SNPs using population-based phasing method Eagle2 [4] with the help of homozygous SNPs. Eagle2 finds haplotypes in the 1000 Genomes haplotype panel that agree with homozygous and heterozygous SNPs, and infers the phasing of heterozygous SNPs based on the haplotype haplotype.

Finally, we construct population-phased allele count matrix Y^0 from the UMI counts for haplotype 1 for each heterozygous SNP and spot, and total allele count matrix D^0 from the sum of UMI counts for both haplotypes.

S2 Copy number probabilistic model for the pure clone case

We model the transcript counts $\mathbf{X}$ using a Negative Binomial distribution and the allele counts $\mathbf{Y}$ using a Beta-binomial distribution. Making a mild assumption that $\mathbf{X}$ and $\mathbf{Y}$ are independent, we obtain $\mathbb{P}(\mathbf{X}, \mathbf{Y} \mid \boldsymbol{\ell}, \mathbf{Z}; \boldsymbol{\mu}, \mathbf{p}, \boldsymbol{\lambda}, \mathbf{D}, (\boldsymbol{\theta}), (W)) = \mathbb{P}(\mathbf{X} \mid \boldsymbol{\ell}, \mathbf{Z}; \boldsymbol{\mu}, \mathbf{p}, \boldsymbol{\lambda}, \mathbf{D}, (\boldsymbol{\theta}), (W))\mathbb{P}(\mathbf{Y} \mid \boldsymbol{\ell}, \mathbf{Z}; \boldsymbol{\mu}, \mathbf{p}, \boldsymbol{\lambda}, \mathbf{D}, (\boldsymbol{\theta}), (W))$. We assume all spots contain cells from either a single clone or normal cells in this section, and generalize the assumption in the next section.

Copy number aberrations affect $\mathbf{X}$ and $\mathbf{Y}$ in the following ways: increasing total copy number leads to increased gene expression and hence higher values in the corresponding entries in $\mathbf{X}$; increase or decrease of copy number of one allele leads to imbalanced read counts between the two alleles and hence the ratio between $\mathbf{Y}$ and $\mathbf{D}$ is biased away from 0.5 at corresponding entries.

Let $T_n = \sum_{g=1}^{G} x_{g,n}$ be the total transcript counts across all genomic bins for the nth spot. Suppose the cells in this spot are all from clone m. We assume the copy numbers at each bin g scale the baseline proportion of transcript counts λ_g by $\frac{a_{g,m}+b_{g,m}}{2}$. We model $x_{g,n}$ by a Negative Binomial distribution parameterized by $T_n, \boldsymbol{\lambda}, \mathbf{A}, \mathbf{B}$ and an additional over-dispersion parameter ϕ:

$$x_{g,n} \mid \ell_n = m \sim NB\left(T_n \frac{\lambda_g(a_{g,m} + b_{g,m})}{\sum_g \lambda_g(a_{g,m} + b_{g,m})}, \phi\right). \quad (1)$$

The Negative Binomial distribution can be viewed as an approximation for the Dirichlet Multinomial distribution $DirMult(T_n, \boldsymbol{\alpha})$, where $\alpha_g \propto \lambda_g \frac{a_{g,m}+b_{g,m}}{2}$. The Multinomial probability parameters are constrained

to be a simplex, and are more difficult to optimize than a Negative Binomial distribution.

We model $\mathbf{Y}$ using a Beta-binomial distribution given the ratio between B allele copy number and total copy numbers at each genomic bin and the total SNP-covering reads $\mathbf{D}$. The Beta distribution prior in the Beta-binomial distribution allows larger variance than a binomial distribution, thus taking into account potential sequencing biases and other unknown factors related to allele imbalance.

$$y_{g,n} \mid \ell_n = m \sim BetaBinom\left(D_{g,n}, \tau \frac{b_{g,m}}{a_{g,m} + b_{g,m}}, \tau \frac{a_{g,m}}{a_{g,m} + b_{g,m}}\right). \tag{2}$$

The mean of the Negative Binomial distribution has a fractional form of $\mathbf{A}, \mathbf{B}$, making a direct optimization of integer $\mathbf{A}$ and $\mathbf{B}$ challenging. We transform $\mathbf{A}$ and $\mathbf{B}$ into latent RDR $\boldsymbol{\mu}$ and BAF $\mathbf{p}$ parameters with K unique values across copy number states. Suppose the bin g in clone m takes the kth copy number state, $z_{g,m} = k$, the corresponding latent RDR and BAF are:

$$\begin{aligned} \mu_k &= \frac{a_{g,m} + b_{g,m}}{\sum_g \lambda_g (a_{g,m} + b_{g,m})} \\ p_k &= \frac{b_{g,m}}{a_{g,m} + b_{g,m}}. \end{aligned} \tag{3}$$

The probabilities of transcript count $x_{g,n}$ and allele count $y_{g,n}$ parameterized by μ_k and p_k are:

$$\begin{aligned} x_{g,n} \mid \ell_n = m, z_{g,m} = k &\sim NB\left(T_n \lambda_g \mu_k, \phi\right) \\ y_{g,n} \mid \ell_n = m, z_{g,m} = k &\sim BetaBinom\left(D_{g,n}, \tau p_k, \tau(1 - p_k)\right). \end{aligned} \tag{4}$$

Note that the denominator of μ_k is a weighted average of total copy numbers along the genome in each clone, and technically takes different values in different clones. But under the assumption that different clones share many common CNA events, specifically when they are close in lineage, we assume the denominators are similar across clones and the K unique RDR values $\boldsymbol{\mu}$ are shared across clones. This transformation (3) is the basis for inferring integer copy numbers (Section S9). Also note that we express the probabilistic model for individual spots, but it is generalizable to a pseudobulk of multiple spots.

Visualization the data: We define *observed RDR* as $\frac{x_{g,n}}{T_n \lambda_g}$ and *observed BAF* as $\frac{y_{g,n}}{D_{g,n}}$ for data visualization. Additionally, if the latent RDR and BAF are found to be close to 1 and 0.5, respectively, along the genome in a clone, we drop this clone for visualization because it does not contain CNAs detectable to CalicoST and likely contains mainly normal cells.

S3 Copy number probabilistic model for the heterogeneous clones case

We derive the probabilistic of transcript counts $\mathbf{X}$ and allele counts $\mathbf{Y}$ allowing each spot to contain a mixture of tumor and normal cells.

Suppose each spot n is a mixture of M clones and a proportion of $\tilde{\theta}_{m,n}$ of the counts are from clone m, $\sum_{m=1}^{M} \tilde{\theta}_{m,n} = 1$. We adapt the mean of the Negative Binomial distribution of $\mathbf{X}$ by a mixture of RDR values. With a little abuse of notation, we denote $\mu_{g,m}$ as the RDR value that bin g in clone m takes among the K unique values, i.e. $\sum_{k=1}^{K} \mathbb{1}[z_{g,m} = k]\mu_{k,m}$.

$$x_{g,n} \sim NB\left(T_n \lambda_g \sum_m \tilde{\theta}_{m,n} \mu_{g,m}, \phi\right). \tag{5}$$

The Beta-binomial distribution of $\mathbf{Y}$ under the mixture is parameterized by

$$y_{g,n} \sim BetaBinom\left(D_{g,n}, \tau\frac{\sum_m \tilde{\theta}_{m,n}\mu_{g,m}p_{g,m}}{\sum_m \tilde{\theta}_{m,n}\mu_{g,m}}, \tau(1-\frac{\sum_m \tilde{\theta}_{m,n}\mu_{g,m}p_{g,m}}{\sum_m \tilde{\theta}_{m,n}\mu_{g,m}})\right). \tag{6}$$

With a similar change in notation of $\mu_{g,m}$ and $p_{g,m}$. See Section S17 for the derivation of (5) and (6).

However, deconvolving the input matrices into multiple cancer clones is challenging and the solutions are potentially non-identifiable. Therefore, we make the simplifying assumption that each spot may only contain at most one cancer clone but can be mixed with normal cells, that is $|\{m \in \text{cancer clones} : \tilde{\theta}_{m,n} > 0\}| \leqslant 1$. Because the RDR and BAF of normal cells are known values, $\mu_{g,m} = 1, p_{g,m} = 0.5$, inferring the RDR and BAF of the only cancer clone in a spot becomes identifiable. We also simplify the notation and define the *tumor count proportion* $\boldsymbol{\theta} = [\theta_n]$ as the proportion of counts from the only cancer clone of each spot if not purely normal, $\theta_n = \sum_{m \in \text{cancer clones}} \tilde{\theta}_{m,n}$. Let $\boldsymbol{\ell} = [\ell_n]$ be the label of the only cancer clone, we rewrite the probabilistic model of $\mathbf{X}$ and $\mathbf{Y}$ under the simplifying assumption:

$$x_{g,n} \mid \ell_n = m \sim NB\left(T_n\lambda_g(\theta_n\mu_{g,m} + 1 - \theta_n), \phi\right) \tag{7}$$

$$y_{g,n} \mid \ell_n = m \sim BetaBinom\left(D_{g,n}, \tau\frac{\theta_n\mu_{g,m}p_{g,m} + 0.5(1-\theta_n)}{\theta_n\mu_{g,m} + 1 - \theta_n}, \tau(1-\frac{\theta_n\mu_{g,m}p_{g,m} + 0.5(1-\theta_n)}{\theta_n\mu_{g,m} + 1 - \theta_n})\right). \tag{8}$$

We take θ_n estimated either using BAF or by cell-type deconvolution methods (e.g., RCTD [5] and CARD [6]) as input assuming a reference of cell-type specific expression is available. Note that we define the proportion $\boldsymbol{\theta}$ as the proportion of counts, instead of the proportion of cells. We claim that the output from cell-type deconvolution methods matches our proportion definition. Different cell types may have different total transcript counts within each cell; by normalizing all cell types to have the same total transcript count in cell-type deconvolution, those methods infer the proportions of counts of each cell type rather than proportions of cells.

S4 Hidden Markov Model (HMM) to infer copy number states

We model $\mathbb{P}(\mathbf{Z})$ using a Markov chain and assume that the copy number states are independent across clones. Thus, $\mathbb{P}(\mathbf{Z}) = \prod_{m=1}^{M} \mathbb{P}(\mathbf{Z}_{\cdot,m})$. Assuming the clone labels $\boldsymbol{\ell}$ are fixed to the last iteration of block coordinate ascent update, we compute $\mathbb{P}(\mathbf{X}, \mathbf{Y} \mid \mathbf{Z}; \boldsymbol{\mu}, \mathbf{p}, \boldsymbol{\ell}, \boldsymbol{\lambda}, \mathbf{D}, (\boldsymbol{\theta}), (W))\mathbb{P}(\mathbf{Z})$ as follows.

Given clone labels $\boldsymbol{\ell}$, we optimize the following objective for $\mathbf{Z}$, $\boldsymbol{\mu}$, and $\mathbf{p}$:

$$\begin{aligned} &\underset{\substack{\boldsymbol{\mu},\mathbf{p}\in\mathbb{R}^K \\ \mathbf{Z}\in\{1,\ldots,K\}^{G\times M}}}{\arg\max} \quad \mathbb{P}(\mathbf{X}, \mathbf{Y} \mid \mathbf{Z}; \boldsymbol{\mu}, \mathbf{p}, \boldsymbol{\ell}, \boldsymbol{\lambda}, \mathbf{D}, (\boldsymbol{\theta}), (W))\mathbb{P}(\mathbf{Z}) \\ = &\underset{\substack{\boldsymbol{\mu},\mathbf{p}\in\mathbb{R}^K \\ \mathbf{Z}\in\{1,\ldots,K\}^{G\times M}}}{\arg\max} \prod_{m=1}^{M}\left(\prod_{n:\ell_n=m} \mathbb{P}(\mathbf{X}_{\cdot,n}, \mathbf{Y}_{\cdot,n} \mid \mathbf{Z}_{\cdot,m}; \boldsymbol{\mu}, \mathbf{p}, \boldsymbol{\lambda}, \mathbf{D}_{\cdot,n}, (\theta_n))\right) \end{aligned} \tag{9}$$

With an abuse of notation, we use $\boldsymbol{\ell}$ to denote the values that the random variable of clone label takes, rather than the random variable itself.

Given that CNAs affect large contiguous regions in the genome, adjacent genomic bins tend to have the same copy number state. We model the copy number states $\mathbf{Z}_{\cdot,m}$ using a Markov model for each clone m

with equal values for the start probability and inter-state transition probabilities:

$$\mathbb{P}(\mathbf{Z}_{\cdot,m}) = \mathbb{P}(z_{1,m}) \prod_{g=2}^{G} \mathbb{P}(z_{g,m} \mid z_{g-1,m}) \tag{10}$$

$$\mathbb{P}(z_{1,m}) = (\frac{1}{K}, \ldots, \frac{1}{K}) \tag{11}$$

$$\mathbb{P}(z_{g,m} \mid z_{g-1,m}) = \begin{cases} t & (z_{g,m} = z_{g-1,m}) \\ \dfrac{1-t}{K-1} & (z_{g,m} \neq z_{g-1,m}) \end{cases} \tag{12}$$

where the parameter t is a user-defined parameter of self-transition probability. The objective (9) is a hidden Markov Model (HMM) under this prior distribution of $\mathbb{P}(\mathbf{Z}_{\cdot,m})$. While the transition probability t can be inferred during HMM inference, $\mathbf{X}$ and $\mathbf{Y}$ tend to have large variances in SRT data; thus, the estimated t tends to favor a high probability of inter-state transition and disagrees with CNA event sizes and frequencies in reality. We use $t = 1 - 10^{-5}$ by default. We use the Baum-Welch algorithm to estimate RDR $\boldsymbol{\mu}$ and BAF $\mathbf{p}$ parameters.

While the MLE estimate of $\mathbf{Z}$ in (9) can be solved by Viterbi algorithm, we instead compute the full posterior distribution of $z_{g,m}$ given by the forward-backward algorithm, which marginalizes $z_{g,m}$ over all possible copy number states of other bins.

$$\hat{z}_{g,m} = \arg\max_{z_{g,m}} \mathbb{P}(z_{g,m} \mid \mathbf{X}_{\cdot,m}, \mathbf{Y}_{\cdot,m})$$

In practice, the counts of individual spots are still sparse and the HMM likelihood optimization may fall into local maxima. On the other hand, the counts aggregated across spots from the same clone are much less sparse and suffer less from the local maxima. Let $\mathbf{x}_m$ be the aggregated transcript counts across spots of clone m, $\mathbf{y}_m$ and $\mathbf{d}_m$ be the aggregated B allele and total allele count of clone m, i.e., $\mathbf{x}_m = \sum_{n:\ell_n=m} X_{\cdot,n}$ and $\mathbf{y}_m$ and $\mathbf{d}_m$ are defined similarly. We showed that the likelihood of $(\mathbf{x}_m, \mathbf{y}_m)$ is only different from that of individual spots, $(\mathbf{X}_{\cdot,n}, \mathbf{Y}_{\cdot,n})$ for all spots n that belong to clone m, by a constant, if dropping the over-dispersion parameters in the probabilistic model (Section S18). Therefore we optimize the following likelihood function.

$$\arg\max_{\boldsymbol{\mu},\mathbf{p}\in\mathbb{R}^K} \prod_{m=1}^{M} \left(\sum_{\mathbf{Z}_{\cdot,m}} \mathbb{P}(\mathbf{x}_m, \mathbf{y}_m \mid \mathbf{Z}_{\cdot,m}; \boldsymbol{\mu}, \mathbf{p}, \boldsymbol{\lambda}, \mathbf{d}_m, (\theta_n), (W)) \mathbb{P}(\mathbf{Z}_{\cdot,m}) \right). \tag{13}$$

S5 Leveraging spatial coherence for inferring clone labels by Hidden Markov Random Field

We assume $\mathbb{P}(\ell; \mathbf{S})$ follows a Markov Random Field, i.e., the clone label ℓ_s of spot s depends on the clone labels of adjacent spots. Assuming the copy number states $\mathbf{Z}$ are fixed to the last iteration of block coordinate ascent update, we optimize the hidden Markov Random Field $\mathbb{P}(\mathbf{X}, \mathbf{Y} \mid \boldsymbol{\ell}; \mathbf{Z}, \boldsymbol{\mu}, \mathbf{p}, \boldsymbol{\lambda}, \mathbf{D}, (\boldsymbol{\theta}), (W))\mathbb{P}(\boldsymbol{\ell}; \mathbf{S})$ to infer $\boldsymbol{\ell}$.

Given an estimated RDR $\boldsymbol{\mu}$ and BAF $\mathbf{p}$ and the most probable values of $\mathbf{Z}$, we optimize the following objective over clone labels $\boldsymbol{\ell}$

$$\begin{aligned} &\arg\max_{\boldsymbol{\ell}\in\{1,\ldots,M\}^N} \mathbb{P}(\mathbf{X}, \mathbf{Y} \mid \boldsymbol{\ell}; \mathbf{Z}, \boldsymbol{\mu}, \mathbf{p}, \boldsymbol{\lambda}, \mathbf{D}, (\boldsymbol{\theta}), (W))\mathbb{P}(\boldsymbol{\ell}; \mathbf{S}) \\ =& \arg\max_{\boldsymbol{\ell}\in\{1,\ldots,M\}^N} \left(\prod_{n} \mathbb{P}(\mathbf{X}_{\cdot,n}, \mathbf{Y}_{\cdot,n} \mid \ell_n; \mathbf{Z}_{\cdot,n}, \boldsymbol{\mu}, \mathbf{p}, \boldsymbol{\lambda}, \mathbf{D}, (\boldsymbol{\theta}), (W)) \right) \mathbb{P}(\boldsymbol{\ell}; \mathbf{S}) \end{aligned} \tag{14}$$

We assume clones are spatially coherent and use a Potts model [7] as the prior probability for $\mathbb{P}(\boldsymbol{\ell}; \mathbf{S})$. Let $E = [e_{n,n'}]$ where $e_{n,n'} \in [0,1]$ be the weighted adjacency matrix, where $e_{n,n'} = 1$ indicates spot n and n' are adjacent with very small distance and $e_{n,n'} = 0$ indicates n and n' are not adjacent (see Section S6 "Constructing weighted adjacency matrix E"). The Potts model gives a higher probability to labelings of vertices where vertices incident to an edge have the same label. This models the assumption that spots containing cancerous cells are more likely to be spatially contiguous. The Potts model describe the prior probability of spot labels $\boldsymbol{\ell}$ by the weighted number of edges with identical labels, $e_{n,n'}\mathbb{1}[\ell_n = \ell_{n'}]$:

$$\log \mathbb{P}(\boldsymbol{\ell}; \mathbf{S}) \propto \sum_{n=1}^{N} \sum_{m=1}^{M} \alpha_m \mathbb{1}[\ell_n = m] + \beta \sum_{1 \leqslant n < n' \leqslant N} e_{n,n'} \mathbb{1}[\ell_n = \ell_{n'}].$$

Note that the weighted adjacency matrix E contains both within-slice spatial adjacency and the alignment W across slices if it is available. The objective (14) is a hidden Markov Random Field (HMRF) and we use iterated conditional modes [8] for optimizing $\boldsymbol{\ell}$.

Notice that we obtain the full posterior probability of $\mathbb{P}(z_{g,m} \mid \mathbf{X}_{\cdot,m}, \mathbf{Y}_{\cdot,m})$ via forward-backward algorithm, and accordingly we give the option in CalicoST to leverage the full posterior probability. Denote the full posterior probability $\mathbb{Q}(\mathbf{Z}_{\cdot,m}) = \prod_g \mathbb{P}(z_{g,m} \mid \mathbf{X}_{\cdot,m}, \mathbf{Y}_{\cdot,m})$, CalicoST can alternatively solve the following objective function that uses $\mathbb{Q}(\mathbf{Z}_{\cdot,m})$:

$$\begin{aligned} &\underset{\boldsymbol{\ell} \in \{1,\ldots,M\}^N}{\arg\max} \left(\prod_{n=1}^{N} \mathbb{P}(\mathbf{X}_{\cdot,n}, \mathbf{Y}_{\cdot,n} \mid \ell_n; \boldsymbol{\lambda}, \mathbf{D}_{\cdot,n}, \theta_n, W, \boldsymbol{\mu}, \mathbf{p}) \right) \mathbb{P}(\boldsymbol{\ell}; \mathbf{S}) \\ = &\underset{\boldsymbol{\ell} \in \{1,\ldots,M\}^N}{\arg\max} \prod_{n=1}^{N} \prod_{m=1}^{M} \left(\sum_{\mathbf{Z}_{\cdot,m}} \mathbb{P}(\mathbf{X}_{\cdot,n}, \mathbf{Y}_{\cdot,n} \mid \mathbf{Z}_{\cdot,m}; \boldsymbol{\lambda}, \mathbf{D}_{\cdot,n}, \theta_n, W, \boldsymbol{\mu}, \mathbf{p}) \mathbb{Q}(\mathbf{Z}_{\cdot,m}) \right)^{\mathbb{1}[\ell_n = m]} \mathbb{P}(\boldsymbol{\ell}; \mathbf{S}). \end{aligned}$$

S6 Constructing the weighted adjacency matrix E for HMRF

For a single slice, we construct a binary adjacency matrix $E = [e_{n,n'}]$, where $e_{n,n'} = 1$ indicates spot n and n' are either adjacent hexagon endpoints for Visium layout, or within the K-nearest neighbors of each other for other spatial layouts.

For K slices from the same tissue, we construct a weighted adjacency matrix E using a block diagonal form

$$\begin{pmatrix} E_1 & W_{1,2} & \cdots & W_{1,K} \\ W_{1,2}^T & E_2 & \cdots & W_{2,K} \\ \vdots & \vdots & \ddots & \vdots \\ W_{1,K}^T & W_{2,K}^T & \cdots & E_K \end{pmatrix},$$

where E_i is the binary adjacency matrix for spots in slice i, and $W_{i,j}$ is either the probabilistic alignment between spots in slice i and slice j or a zero matrix if the alignment between the two slices is unavailable. For the multi-slice samples analyzed here, we obtained $W_{i,j}$ from the probabilistic alignment matrix output from PASTE2 [9] Specifically, PASTE2 outputs a probabilistic alignment matrix $W_{i,j}^0$ whose entries are non-negative entries and sum to 1. Larger values in $W_{i,j}^0$ correspond to higher probabilities of alignment of spots i and j. We scaled PASTE2 alignment matrix by $W_{i,j} = \frac{W_{i,j}^0}{\max W_{i,j}^0}$, that is, the scaled $W_{i,j}$ has a max value of 1, which is consistent with the binary adjacency matrix for within-slice adjacency. Because of this scaling, the matrix E gives equal weight to within-slice adjacency and between-slice adjacency. If slice i and j are distant in space or are not aligned (e.g. multiple distinct 2D slices from a larger tissue slice as in the multi-section prostate cancer data), then we use a zero matrix for $W_{i,j}$. We denote the list of all alignment matrices as $W = [W_{i,j}]$, which is an input to CalicoST's objective function in equation (1).

S7 Identifying a confident set of normal spots and obtaining baseline transcript counts $\boldsymbol{\lambda}$

The Negative Binomial model of transcript counts $\mathbf{X}$ depends on the baseline proportions of transcript counts $\boldsymbol{\lambda}$ in the diploid normal genome. We identify spots that contain mostly diploid normal cells and derive λ as follows.

If the tumor count proportions θ_n are available, for example by CalicoST, we select the set of spots for which θ_n is below a threshold as confident normal spots. The threshold is chosen such that the transcript counts of each genomic bin on average is at least 200 when aggregated across confident normal spots.

If each spot is assumed to contain purely normal cells or a single clone, CalicoST identifies a confident set of normal spots with the closest-to-balanced BAF along the genome. It is challenging to evaluate for individual spots whether the BAF is close to balance because total allele counts are sparse. Therefore, we group spots with similar BAF signals along the genome and evaluate the BAF of each group. We infer the grouping by solving the **copy number state and clone inference problem** using only the allele count matrices $\mathbf{Y}, \mathbf{D}$. Let $\mathbf{p}^0 \in \mathbb{R}^K$ be the estimated BAF across K states, and $\mathbf{Z}^0 = [z^0_{g,m}]$ be the state indicator for each genomic bin in each group. Hence, normal spots must be within the group m' with the most balanced BAFs $m' = \arg\min_m \sum_{g=1}^{G} | \sum_{k=1}^{K} \mathbb{1}[z_{g,m} = k] p^0_k - 0.5|$. To avoid the case where a small cancer clones are mixed within spot group m', we select a given number of spots with the smallest variance of log-transformed transcript counts along genome inside clone m' as confident normal spots.

Let J denote the set of confident normal spots, the transcript count proportions across genomic bins in normal cells $\boldsymbol{\lambda}$ are derived by averaging the expression across confident normal spots J and normalizing to sum to 1, $\lambda_g \propto \sum_{i \in J} x_{g,i}$.

S8 Filtering genes and bins based on normal spots

Before solving the **copy number state and clone inference problem**, we remove genomic bins and genes from the transcript count matrix $\mathbf{X}$ and allele count matrix $\mathbf{Y}$ that are unlikely to be dominantly affected by CNAs, using the following procedure.

Since allele imbalance can also be caused by allele-specific gene expression, we aim to remove genomic bins that are potentially affected by allele-specific expression. We assume allele-specific expression affects both tumor and normal cells and identify genomic bins with allele-specific expression if the BAF values in normal spots deviate substantially from 0.5. Let J be the confident set of normal cells and denote the CDF of a Beta-binomial distribution with d total allele counts, y B allele counts, p B allele frequency, and τ over dispersion as $F_{\text{Betabinom}}(y; d, p, \tau)$. The set of genomic bins that are affected by allele-specific expression is

$$\{g \in G : F_{\text{Betabinom}}(\sum_{i \in J} y_{g,i}; \sum_{i \in J} d_{g,i}, 0.5, \tau) < \text{threshold or}$$
$$F_{\text{Betabinom}}(\sum_{i \in J} y_{g,i}; \sum_{i \in J} d_{g,i}, 0.5, \tau) > 1 - \text{threshold}\}$$

We remove these genomic bins in this set from matrices $\mathbf{Y}$ and $\mathbf{D}$.

Differential gene expression not resulting from CNAs can dominate the estimation of RDR $\boldsymbol{\mu}$ when the transcript count of the corresponding gene is high. To reduce this issue, we remove highly expressed genes with large expression fold change between confident normal spots and tumor ones, and between confident normal spots and other spots in initial clone m' if (1) their total transcript counts across all spots

are above the 80% quantile and (2) the log fold change of expression is above a threshold. We argue that CalicoST can still accurately estimate $\boldsymbol{\mu}$ after removing the above set of genes. Because large CNAs alter the expression of many adjacent genes along genome, the remaining genes are sufficient to identify copy number gains or losses.

S9 Inferring allele-specific integer copy numbers per clone

CalicoST estimates the integer allele-specific copy numbers $\mathbf{A}$ and $\mathbf{B}$ using the estimated RDR $\boldsymbol{\mu}$, BAF $\mathbf{p}$, and copy number states $\mathbf{Z}$. Because the RDR and BAF correspond to the transformation from $\mathbf{A}$ and $\mathbf{B}$ in equation (3), we optimize over $\mathbf{A}$ and $\mathbf{B}$ to minimize the deviation between the transformed RDR and BAF values and the estimated ones in (2).

Since $\mathbf{Z}$ groups the genome bins by copy number states, we estimate a single allele-specific copy number for the state within each clone m. We introduce the notation $\tilde{\mathbf{A}} = [\tilde{a}_{k,m}]$ for the K values of A copy and $\tilde{\mathbf{B}} = [\tilde{b}_{k,m}]$ for the K values of B copy for each clone m. Given the copy number states $\mathbf{Z}$, the allele-specific copy numbers $\mathbf{A}, \mathbf{B}$ along the entire genome can be expressed using $\tilde{\mathbf{A}}, \tilde{\mathbf{B}}$:

$$a_{g,m} = \sum_{k=1}^{K} \mathbb{1}_{z_{g,m}=k} \tilde{a}_{k,m}.$$

In addition, the transformation to RDR and BAF from $\tilde{\mathbf{A}}, \tilde{\mathbf{B}}$ is

$$\begin{aligned} \mu_k &= \frac{\tilde{a}_{k,m} + \tilde{b}_{g,m}}{\sum_{k=1}^{K} \tilde{\lambda}_{k,m} (\tilde{a}_{k,m} + \tilde{b}_{k,m})} \\ p_k &= \frac{\tilde{b}_{k,m}}{\tilde{a}_{k,m} + \tilde{b}_{k,m}}, \end{aligned}$$

where $\tilde{\lambda}_{k,m} = \sum_g \mathbb{1}_{z_{g,m}=k} \lambda_g$.

Generally, the integer copy numbers are not identifiable; different $\tilde{\mathbf{A}}, \tilde{\mathbf{B}}$ may lead to the same values of $\boldsymbol{\mu}, \mathbf{p}$, for example, scaling both $\tilde{\mathbf{A}}$ and $\tilde{\mathbf{B}}$ by the same integer multiple. Therefore, we constrain the allele-specific integer copy numbers not to be too large and the ploidy of the genome ψ_m not to be too high. Denoting the constrained space as $\mathcal{C}$, we solve the following constrained optimization problem for each clone m to infer $\tilde{\mathbf{A}}_{\cdot,m}$ and $\tilde{\mathbf{B}}_{\cdot,m}$:

$$\begin{aligned} \underset{(\psi_m, \tilde{\mathbf{A}}_{\cdot,m}, \tilde{\mathbf{B}}_{\cdot,m}) \in \mathcal{C}}{\arg\min} & \left(\sum_{k=1}^{K} \left(w_k^{\text{RDR}} |\mu_k - \frac{\tilde{a}_{k,m} + \tilde{b}_{g,m}}{\psi_m}|^2 + w_k^{\text{BAF}} |p_k - \frac{\tilde{b}_{k,m}}{\tilde{a}_{k,m} + \tilde{b}_{k,m}}|^2 \right) \right) + \\ & w^{\text{ploidy}} \|\psi_m - \sum_{k=1}^{K} \tilde{\lambda}_{k,m} (\tilde{a}_{k,m} + \tilde{b}_{k,m})\|_2^2, \end{aligned} \tag{15}$$

where w_k^{RDR}, w_k^{BAF} and w^{ploidy} are weights.

Different weights lead to different optima in allele-specific integer copy numbers. In CalicoST, we implemented two settings of the weights and constraints. In the first setting, we set $w^{\text{ploidy}} = \infty$, $w_k^{\text{RDR}} = 0.3 w_k^{\text{BAF}} = \sum_{g=1}^{G} \mathbb{1}[z_{g,m} = k]$, where we applies a scaling of w_k^{RDR} to account for the different dynamic range of RDR (ranging from 0 to an integer total copy number divided by 2) and BAF (ranging from 0 to 1). We set the constrained space $\mathcal{C}$ as $\mathcal{C}$ as $\mathcal{C} = \{\tilde{a}_{k,m} \leqslant 4 (\forall k), \tilde{b}_{k,m} \leqslant 4 (\forall k), \psi_m < 3\}$.

In the second setting, we borrow the idea from CHISEL [10] and identify a balanced diploid state j such that estimated BAF p_j is close to 0.5 and estimated RDR μ_j is the lowest. The balanced diploid state will have integer copy numbers $\tilde{a}_{j,m} = \tilde{b}_{j,m} = 1$. Using the balanced diploid state, we set the constrained space as $\mathcal{C}$ as $\mathcal{C}$ as $\mathcal{C} = \{\tilde{a}_{j,m} = \tilde{b}_{j,m} = 1, \tilde{a}_{k,m} \leqslant 4(\forall k), \tilde{b}_{k,m} \leqslant 4(\forall k), \psi_m < 3\}$, and set $w^{\text{ploidy}} = 0$, $w_j^{\text{RDR}} = \infty$, and $w_k^{\text{RDR}} = 0.3 w_k^{\text{BAF}} = \sum_{g=1}^{G} \mathbb{1}[z_{g,m} = k]$ for the remaining k as above.

Note that CHISEL's objective function for estimating integer copy numbers is a slight variation of (15). In CHISEL, w^{ploidy} is set to 0. CHISEL models observed RDR and BAF using Gaussian distributions and uses the inverse of Gaussian variance as w_k^{BAF} and w_k^{RDR}. In addition, instead of constraining to space $\mathcal{C}$, CHISEL incorporates a penalty term of ψ_m. Because the gene expression in SRT has higher variance than the read coverage in scDNA-seq, there may not be a robust penalty weight for ψ_m across various SRT samples. Therefore, we enforce the constraint $\mathcal{C}$ in CalicoST instead of using penalties.

S10 Choosing the number of clones

CalicoST requires an initial number of clones provided by users and uses a statistics to evaluate the similarity of CNA profiles and combine initial clones. It is challenging to apply existing model selection criteria such as AIC and BIC to our model, which has a complicated parameter structure with an HMM model nested inside an HMRF. Instead, we evaluate the similarity between CNA profiles of two clones using the following statistics and merge them if their similarity passes a threshold.

Given two clones m and m' with the corresponding hidden states $\mathbf{Z}_{\cdot,m}$ and $\mathbf{Z}_{\cdot,m'}$ in the HMM, we suppose their hidden states differ in I intervals of genomic bins, $R_1, \ldots, R_I$, where each interval has a consistent copy number state within each clone. Let $\mathbf{Z}_{R_i,m} = (z_{g,m})_{g \in R_i}$ and $\mathbf{Z}_{R_i,m'} = (z_{g,m'})_{g \in R_i}$ be the hidden states of clone m and m' restricted to genomic interval R_i; they are constant vectors by our construction of R_i. If the CNAs of clone m and m' are similar in interval R_i, the probabilities of observing the count data under copy number states $\mathbf{Z}_{R_i,m}$ should be similar to those under copy number states $\mathbf{Z}_{R_i,m'}$. We use the Neyman Pearson statistics, i.e., the ratio between the probabilities under the two copy number states, to indicate the similarity of copy numbers of the two clones at R_i:

$$T_{m,m',R_i} = \frac{\mathbb{P}(\mathbf{X}_{R_i,m}, \mathbf{Y}_{R_i,m} \mid \mathbf{Z}_{R_i,m}, \boldsymbol{\mu}, \mathbf{p})\mathbb{P}(\mathbf{X}_{R_i,m'}, \mathbf{Y}_{R_i,m'} \mid \mathbf{Z}_{R_i,m'}, \boldsymbol{\mu}, \mathbf{p})}{\mathbb{P}(\mathbf{X}_{R_i,m}, \mathbf{Y}_{R_i,m} \mid \mathbf{Z}_{R_i,m'}, \boldsymbol{\mu}, \mathbf{p})\mathbb{P}(\mathbf{X}_{R_i,m'}, \mathbf{Y}_{R_i,m'} \mid \mathbf{Z}_{R_i,m}, \boldsymbol{\mu}, \mathbf{p})}.$$

$T_{m,m',R_i} \approx 1$ indicates the probabilities are indeed similar and the CNAs of clone m in interval R_i are similar to those of clone m'; while $T_{m,m',R_i} \gg 1$ indicates CNAs of clone m largely differ from those of clone m' in interval R_i. CalicoST decides to merge a set of initial clones if the CNAs of all pairs of clones are similar across all intervals, $T_{m,m',R_i} \leqslant 1 + \epsilon$ for all clone pairs m, m' for all intervals R_i under a user-defined threshold ϵ.

S11 Running HATCHet2 on matched WES data of HTAN samples

We ran HATCHet2 (commit 01541aff0dce1377127120b7de92c1f494e112ae) on matched whole exome sequencing (WES) data for eleven HTAN patients (Fig 5). The matched WES data include one sample from healthy tissue and one tumor sample from the bulk tumor section adjacent to section(s) used in spatial transcriptomic analysis. We utilized the 1000G Phase3 SNP panel to identify heterozygous SNPs in HATCHet2. We set HATCHet2's parameter, minimum SNP covering reads, to 100, obtaining variable width bins with a median size of 32kbp. In HATCHet2's hidden Markov model, the number of hidden states was automatically selected from a set range of $[10, 15]$. HATCHet2 deconvolves the WES data into multiple clones, especially when there are multiple WES samples from the same tumor, and selects the best number of

clones. In all samples except HT253C1 and HT268B1, the number of cancer clones was automatically selected from the range [1,2] and the best solution had one cancer clone. For HT253C1 and HT268B1, the number of tumor clones was set to two to produce a more accurate purity estimate. HATCHet2 failed to identify any CNAs on two PDAC patients (HT270P1 and HT288P1) as the tumor purities are low.

We used the allele-specific copy numbers inferred by HATCHet2 to evaluate the predictions from CalicoST. Even though the CNAs inferred from WES data may still contain errors, the conclusions drawn from the high agreement between copy numbers inferred by HATCHet2 and CalicoST provide confidence since the two methods employ distinct methodologies and data types (WES and spatial transcriptomics, respectively). Additionally, we found the predictions from HATCHet2 are reliable after manual review (Fig 5). For example, the light blue color (chr4) in HT112C1 represents allele-specific copy number of (1,1) with the BAF value being 0.5, and the orange color (chr3p) represents (2,0), which has the same total copy number as (1,1) but is an LOH supported by the BAF deviation from 0.5. The other allele-specific copy numbers are also strongly supported by the RDR and BAF signals, supporting the reliability of the inferred CNAs.

S12 Evaluating the accuracy of inferred copy numbers

We used three metrics to evaluate the inferred allele-specific integer copy numbers: exact match, precision, and recall. Given G genomic bins, the inferred allele-specific copy numbers $(\hat{a}_g, \hat{b}_g)$ at bin g, and the ground truth allele-specific copy numbers (a_g, b_g), the exact match is the proportion of genomic bins where the inferred allele-specific copy numbers match the ground truth:

$$\frac{1}{G}\left(\sum_{g=1}^{G} \mathbb{1}[\hat{a}_g = a_g \text{ and } \hat{b}_g = b_g]\right).$$

The precision is the proportion of predicted genomic bins with CNAs that are supported by the ground truth, where a change of copy number in either A copy or B copy indicates the existence of CNA:

$$\frac{\sum_g \mathbb{1}[\hat{a}_g \neq 1 \text{ or } \hat{b}_g \neq 1] \times \mathbb{1}[a_g \neq 1 \text{ or } b_g \neq 1]}{\sum_g \mathbb{1}[\hat{a}_g \neq 1 \text{ or } \hat{b}_g \neq 1]}.$$

The recall is the proportion of genomic bins with CNAs that are predicted:

$$\frac{\sum_g \mathbb{1}[\hat{a}_g \neq 1 \text{ or } \hat{b}_g \neq 1] \times \mathbb{1}[a_g \neq 1 \text{ or } b_g \neq 1]}{\sum_g \mathbb{1}[a_g \neq 1 \text{ or } b_g \neq 1]}.$$

We extended the exact match to evaluate inferred copy number states (e.g. amplification state, deletion state) without integer copy numbers. With an abuse of notation, we denote $\hat{z}_g$ (z_g) as the inferred (ground truth) copy number states at bin g. The exact match of copy number states is

$$\frac{1}{G}\left(\sum_{g=1}^{G} \mathbb{1}[\hat{z}_g = z_g]\right).$$

Numbat predicts $\hat{z}_g$ to be one of six copy number states: imbalanced amplification (amp), balanced amplification (bamp), balanced copy number neutral (neu), copy number neutral loss of heterozygosity (cnloh), imbalanced deletion (del), and balanced deletion (bdel). STARCH predicts $\hat{z}_g$ to be one of the three copy number states: amplification (amp), neutral (neu), and deletion (del). We converted the allele-specific integer copy numbers inferred by CalicoST or from WES to these states and compared the exact match values with the other methods.

S13 Evaluating spatial coherence of tumor clones by joincount

We use joincount statistics [11, Chapter 3] to evaluate the spatial coherence of each inferred cancer clone. Joincount statistics describes the spatial autocorrelation of binary data. Given a weighted graph $G = (V, E, W)$ where W is the weighted adjacency matrix, and let $\boldsymbol{\ell} \in \{0,1\}^{|V|}$ be the vertex label, the joincount statistics is the number of edges for which the two endpoints have labels $\{a, b\}$:

$$J_{ab} = \sum_{e=(u,v)\in E} W_{u,v} \mathbb{1}_{\{\ell_u, \ell_v\}=\{a,b\}}.$$

The z-score of joincount describes whether the number of edges is larger or smaller than the expectation assuming labels of the endpoints of each edge are i.i.d. samples from a Bernoulli distribution.

$$\text{z-score}(J_{ab}) = \frac{J - (\sum_{u',v'} W_{u',v'})\mathbb{E}(\mathbb{1}_{\{\ell_u,\ell_v\}=\{a,b\}})}{\sqrt{\sum_{u',v'} W_{u',v'}^2}\text{Std}(\mathbb{1}_{\{\ell_u,\ell_v\}=\{a,b\}})}$$

The higher the z-score of J_{ab} for $a = b$, the more spatially coherent the data is. Let P_a and P_b be the probability of a and b ($a, b \in \{0, 1\}$) in the Bernoulli distribution. The expectation and standard deviation is given by

$$\mathbb{E}(\mathbb{1}_{\{\ell_u,\ell_v\}=\{a,b\}}) = \begin{cases} P_a P_b \ (a = b) \\ 2P_a P_b \ (a \neq b) \end{cases}$$

$$\text{Std}(\mathbb{1}_{\{\ell_u,\ell_v\}=\{a,b\}}) = \begin{cases} \sqrt{(1 - P_a P_b)(P_a P_b)} \ (a = b) \\ \sqrt{(1 - 2P_a P_b)(2P_a P_b)} \ (a \neq b) \end{cases}$$

When there are multiple tumor clones, we compute the z-score of joincount, z-score(J_{11}), for each clone by binarizing clone labels into whether each spot is in the given clone.

S14 Details of CalicoST analysis on Slide-tags data

CalicoST identifies three cancer clones with distinct allele-specific copy numbers (Fig 7a). The three cancer clones occupy the top, bottom left, and bottom right of the space respectively. Comparing CalicoST-inferred clones to the cell types derived from expression in the original publication [12], CalicoST successfully distinguishes between normal and cancer clones and between the two main tumor expression clusters (Fig 7b). In contrast to the analysis in the original publication [12] – which performed an analysis of total copy numbers using InferCNV – CalicoST separates the tumor cells in the bottom part of the slice into two clones. The two clones are distinguished by a LOH event at the end of chr3 (Fig 7c), which is supported by a strong imbalance in the BAF (Fig 7d).

S15 Details of simulated data

S15.1 Simulation based on scRNA-seq data

Since ground truth copy number aberrations for cancer clones are not available in real SRT datasets, we evaluated the performance of CalicoST, STARCH, and Numbat on simulated data. For each simulation, we generated a simulated slice containing 3000 spots on a hexagonal grid which mimics the layout of spots

on the Visium platform. We partitioned the spots into four regions: one region of normal cells and three regions containing distinct cancer clones, each containing truncal CNAs (shared by all clones) and CNAs unique to each clone. In each simulated slice, we assign spots to regions by choosing a random spot as the center of each region and assigning each spot to the region with the closest distance (Fig 11a). We simulate allele-specific CNAs for each clone with the following numbers of truncal/unique CNAs: 1/2, 3/3, and 6/3 corresponding to 3, 6, and 9 total CNAs in a slice, respectively. The length of each CNA is selected from 10Mb, 30Mb, or 50Mb, and for each CNA, we randomly select allele-specific copy numbers of (1,0), (2,0), (2,1), (2,2), or (3,1). We simulated 10 replicates for each setting of the number of CNAs and the length of CNAs.

To simulate gene expression within each spot, we first sample the total UMI count per spot from a Poisson-LogNormal distribution where the parameters are chosen according to the observed UMI counts in Visium data (LogNormal mean of 8.0 and standard deviation of 0.4, see the histogram in Fig 11b). We simulated per-gene UMI counts based on snRNA-seq data from patient HT112C1(HTAN cohort), for which the annotated cell types are available. For each normal spot, we simulate the gene expression count vector by sampling from a Dirichlet Multinomial distribution parameterized by a mixture of snRNA-seq normal cell types, i.e., the Dirichlet Multinomial parameter is $p = \sum_k \beta_k \mu_k$ with β_k as the proportion of cell type k and μ_k is the average gene expression of cell type k in the snRNA-seq data. For tumor gene expression, we cannot directly use the gene expressions in the real data due to the lack of ground truth CNAs. Therefore, we simulate the tumor expression vector from a normal cell type origin by adding differential expressions that are induced by simulated CNAs (fixed fold-change corresponding to the simulated copy numbers) and by other regulatory mechanisms (assuming 30% of genes are differentially expressed between tumor and normal and each gene has a uniformly randomly sampled log fold change). We selected 30% because at this percentage we observed a clear separation between simulated normal and tumor spots in a 2-dimensional UMAP projection.

We also simulated SNP-covering UMIs based on the SNP locations in the same patient. We sampled the total SNP-covering UMIs per spot from a PoissonLogNormal distribution with a LogNormal mean of 6 and standard deviation of 0.4 to match the median SNP-covering UMIs per spot observed in real data (Fig 11c). Using the total number of SNP-covering UMIs in a spot, we sample the per-SNP UMI from a Dirichlet Multinomial distribution with equal Multinomial probabilities across loci, leading to a simulated D^0 matrix in the Method Section. We further simulated the population-phased allele count matrix Y^0 by a Beta-binomial distribution from D^0, where the mean of Beta-binomial is the proportion of B copies across all copies of the simulated CNAs. Note that in the simulations the population-based phasing does not have phase switch errors; however, both Numbat and CalicoST are run in their standard settings which will attempt to correct errors in the population-based phasing.

CalicoST achieves the highest accuracy in identifying cancer clones evaluated by adjusted rand index (ARI) under all settings of the simulation (Fig 11d) with an average ARI of 0.87, compared to 0.34 of Numbat and 0.50 of STARCH. CalicoST also more accurately identifies CNAs of different types, achieving an average F1 score of 0.52 in identifying CNLOH events across all simulations, which can only be identified by allele-specific copy number methods – compared to an average F1 = 0.18 for Numbat (Fig 11e). As Numbat does not use spatial information while STARCH is not allele-specific, CalicoST achieves a higher performance than both methods by combining the spatial and allele information.

S15.2 Simulation based on WES data

For each simulation, we generated a simulated slice containing 3000 spots on a hexagonal grid and partitioned the space into two parts: containing only normal cells, and containing a tumor-normal admixture

We varies the fractions (25%, 50%, 75%) of space to be tumor-normal admixture and denoted as tumor fraction for simplicity (Fig 12a for example for 50% tumor fraction). We simulated the transcript counts, B allele counts, and total allele counts of each normal (resp. tumor) spot by down-sampling from WES data from a normal (resp. tumor) sample in HTAN. Specifically, given the read counts from WES per genomic bin of a normal/tumor sample, we sampled reads from the WES data for each spot to obtain a total transcript count following a PoissonLogNormal distribution with LogNormal mean 8 and standard deviation 0.4 . Similarly, we sampled the allele count in WES by a given ratio to simulate allele counts for each spot such that the total allele count matched a random number we sampled from the PoissonLogNormal distribution with LogNormal mean 6 and standard deviation 0.4. Note that the bulk tumor samples of WES contain a mixture of normal and cancer cells. We used one of the three HTAN samples which contained a single cancer clone in HATCHet2 copy number analysis (HT339B1 breast cancer sample, HT253C1 colorectal liver-met sample, and HT230C1 colorectal liver-met sample) for each slice, and simulated five replicates for each HTAN sample for each tumor fraction.

We estimated the tumor proportion θ for each spot using CalicoST according to the procedure described in Section Inferring tumor proportions using BAF (Fig 12b). We then ran CalicoST using the estimated value θ to infer CNAs and cancer clones. CalicoST achieves 76%–92% accuracy when compared to the allele-specific copy number calls on WES data obtained by HATCHet2 (Fig 12c). The accuracy matches the accuracy of CalicoST when applied to Visium samples in Fig 2a, further demonstrating the reliability of CalicoST's accuracy. In addition, we evaluated the ability of CalicoST to distinguish between spots containing only normal cells and containing tumor-normal admixture. We used AUC to evaluate the agreement between CalicoST-inferred tumor proportion and the simulated spot labels. CalicoST-inferred tumor proportions accurately distinguish between the two types of spots with AUC near 1 (Fig 12d).

We next simulated a more complicated geometry: we partitioned the grid into 10-by-6 equally-sized blocks (50 spots per block) or 20-by-12 blocks (on average 12.5 spots per block) and assigned each block to be tumor-normal admixture with probability 0.25, 0.5, and 0.75, corresponding to the three tumor fractions (Fig 12e,i for examples with 50% tumor). We simulated the counts under each geometry by down-sampling WES samples in the same way as described above, and applied CalicoST to infer tumor proportions (Fig 12f,j) and allele-specific CNAs. We observed that when tumor fraction is 50% and 75% under this complex geometry, CalicoST's accuracy in inferring CNAs remains comparable with the previous simulation and the accuracy of distinguishing between spots containing only normal cells and containing tumor-normal admixture is nearly perfect (AUC near 1, Fig 12g,h,k,l). In the most challenging scenario with 25% tumor fraction, CalicoST has a decreased accuracy with an average of 0.61 for 10-by-6 blocks and 0.59 for 20-by-12 blocks (Fig 12 g,h,k,l).

S16 Running Numbat, STARCH, and InferCNV on HTAN and prostate cancer samples

We ran STARCH, Numbat, and InferCNV on each slide separately to avoid batch effects. We tune the Numbat's parameters, `max_entropy` and `min_LLR`, to obtain better results: we start with the recommended value of 0.8 for `max_entropy` and default value 5 for `min_LLR`, but if it cannot identify any CNA event, we increase `max_entropy` to 0.95 and decrease `min_LLR` to 2. Table 1 includes the Numbat configurations and final status across slices.

A required input for InferCNV is an annotation of each spot as tumor or normal. SpatialInferCNV [13] provides a GUI that allows the user to manually select spots/regions for copy number analysis with InferCNV. SpatialInferCNV requires users to explicitly call InferCNV, as noted in its tutorial (https://aerickso.github.

io/SpatialInferCNV/). Thus, SpatialInferCNV does not have any additional capabilities for inferring CNAs beyond those provided by InferCNV once the spots/regions are selected. Thus, we included InferCNV and not SpatialInferCNV in our comparison. In our analysis using InferCNV, we used a manual tumor/normal annotation that is based on manual review of H&E images [14]. This tumor/normal annotation serves a similar function as the spot/region selection module of SpatialInferCNV.

Table 1: Running configuration of Numbat

Slice	Configuration	Status
CRC liver metastasis all slices	recommended	Finished
prostate H1_2, H1_4	`min_LLR`=2, `max_entropy`=0.95	No CNA detected
prostate H1_5	`min_LLR`=2, `max_entropy`=0.95	Finished
prostate H2_1, H2_5	recommended	Finished

S17 Derivation of the probabilistic model for the heterogeneous clone case

S17.1 Negative Binomial model for X

We use a Negative Binomial distribution to empirically approximate a Dirichlet-Multinomial distribution for expression counts. We start with deriving the Dirichlet-Multinomial distribution first. We use the following notations defined previously: total UMI counts T_n, the probability $\boldsymbol{\lambda}$ of sequencing a UMI in normal cells, the proportion $\tilde{\theta}_{m,n}$ of UMIs from clone m, A and B allele copy numbers per bin per clone $\mathbf{A} = [a_{g,m}], \mathbf{B} = [b_{g,m}] \in \mathbb{Z}^{G \times M}$. Let the probability of sequencing a UMI at each bin in cell mixture of be $\boldsymbol{\alpha} \in \mathbb{R}^G$, which is the parameter in the Dirichlet-Multinomial distribution $DirMult(T, \boldsymbol{\alpha})$.

$$\begin{aligned} \alpha_g &\propto \sum_m \theta_{m,n} \mathbb{P}(\text{sequencing a UMI from bin } g \mid \text{clone } m) \\ &= \sum_m \theta_{m,n} \frac{\lambda_g (a_{g,m} + b_{g,m})}{\sum_{g'} \lambda_{g'} (a_{g',m} + b_{g',m})}. \end{aligned}$$

Using the definition of RDR $\mu_{g,m} = \frac{a_{g,m}+b_{g,m}}{\sum_g \lambda_g (a_{g,m}+b_{g,m})}$, we simplify $\boldsymbol{\alpha}$ by

$$\alpha_g \propto \sum_m \theta_m \lambda_g \mu_{g,m}.$$

α_g should satisfy $\sum_{g'} \alpha_{g'} = 1$, and we can verify that $\sum_g \sum_m \theta_m \lambda_g \mu_{g,m} = 1$. Therefore,

$$\alpha_g = \sum_m \theta_m \lambda_g \mu_{g,m}.$$

As a result, the Negative Binomial distribution that approximates the Dirichlet-Multinomial distribution is

$$NB(T\alpha_g, \phi) = NB(T\lambda_g \sum_m \theta_m \mu_{g,m}, \phi).$$

S17.2 Beta-binomial model for Y

In a mixture of clones with UMI proportion per clone $\theta_{m,n}$ and clone-specific BAF $p_{g,m}$, the mixture BAF in the Beta-binomial distribution (5) is

$$\begin{aligned}\text{mixture BAF} &= \frac{\sum_m \theta_{m,n}\mathbb{P}(\text{sequencing a UMI from bin } g \mid \text{clone } m)p_{g,m}}{\sum_m \theta_m \mathbb{P}(\text{sequencing a UMI from bin } g \mid \text{clone } m)} \\ &= \frac{\sum_m \theta_{m,n}\frac{\lambda_g(a_{g,m}+b_{g,m})}{\sum_{g'}\lambda_{g'}(a_{g',m}+b_{g',m})}p_{g,m}}{\sum_m \theta_{m,n}\frac{\lambda_g(a_{g,m}+b_{g,m})}{\sum_{g'}\lambda_{g'}(a_{g',m}+b_{g',m})}} \\ &= \frac{\sum_m \theta_{m,n}\lambda_g\mu_{g,m}p_{g,m}}{\sum_m \theta_{m,n}\lambda_g\mu_{g,m}} \\ &= \frac{\sum_m \theta_{m,n}\mu_{g,m}p_{g,m}}{\sum_m \theta_{m,n}\mu_{g,m}}\end{aligned}$$

S17.3 Relationship between tumor UMI proportion θ_n, tumor purity ρ, and cancer cell fraction (CCF)

Whole genome sequencing (WGS) technology measures DNA of mixed clones. Previous methods [15, 16] for studying single-nucleotide variants (SNVs) and CNAs on WGS data express RDR and BAF in the bulk mixture using the *tumor purity* and *cancer cell fraction (CCF)*. Tumor purity ρ is the fraction of tumor cells in the bulk of cells. CCF is the fraction of tumor cells carrying a given somatic mutation. Under our assumption that the bulk contains one tumor clone besides normal cells, CCF=1. We denote A allele copy number as $\mathbf{A} \in \mathbb{Z}^G$ and B allele copy number $\mathbf{B} \in \mathbb{Z}^G$ for the tumor clone. And the normal clone has one A copy and one B copy for the entire genome. Previous work has the following expression of a BAF of the bulk of cells at bin g:

$$\text{bulk BAF} = \frac{\rho b_g + (1-\rho)}{\rho(a_g + b_g) + 2(1-\rho)}. \tag{16}$$

We use tumor UMI proportion θ_n to express RDR and BAF in the bulk mixture of SRT data in Section S3. Our BAF formula in equation (8) naturally extends to WGS. Specifically θ_n now represents the proportion of reads rather than UMIs from tumor cells, and RDR μ_g the tumor clone is a function of copy number of bin length l_g of bin g and total genome length $L = \sum_g l_g$, $\mu_g = \frac{a_g+b_g}{\sum_g l_g/L(a_g+b_g)}$. For simplicity, we drop the subscription that specifies spot n. Then our formula for bulk BAF extended to WGS is

$$\text{bulk BAF} = \frac{\theta\mu_g p_g + 0.5(1-\theta)}{\theta\mu_g + (1-\theta)}. \tag{17}$$

We will show that our formula and (16) are equivalent in the next.

Theorem 1. *The bulk BAF expressions in equation* (16) *and in equation* (17) *are equivalent in WGS data.*

The key to proving the equivalence is the relationship between tumor purity ρ and tumor read proportion θ. WGS data satisfies the following probability in generating sequencing reads, through which θ can be expressed by ρ.

Property 1. *Given tumor purity ρ, the normal genome that is partitioned into G bins, the length l_g of bin g, the probability of sequencing read r coming from tumor (or normal) cells is proportional to the fraction of*

basepairs from tumor (or normal) genome.

$$\mathbb{P}(r \in \textit{tumor}) \propto \rho \sum_{g=1}^{G} (a_g + b_g) l_g$$
$$\mathbb{P}(r \in \textit{normal}) \propto \rho \sum_{g=1}^{G} 2 l_g.$$

Therefore, the tumor read proportion θ is

$$\theta = \frac{\mathbb{P}(r \in \text{tumor})}{\mathbb{P}(r \in \text{tumor}) + \mathbb{P}(r \in \text{normal})} = \frac{\rho \sum_g (a_g + b_g) l_g}{\rho \sum_g (a_g + b_g) l_g + (1-\rho) \sum_g 2 l_g}$$

Proof of Theorem 1. Dividing the numerator and denominator of θ by L gives $\theta = \frac{\frac{1}{L}\rho \sum_g (a_g+b_g) l_g}{\frac{1}{L}\rho \sum_g (a_g+b_g) l_g + \frac{1}{L}(1-\rho)\sum_g 2l_g}$. Also let the denominator of θ be Denom $= \frac{1}{L}\rho \sum_g (a_g + b_g) l_g + \frac{1}{L}(1-\rho)\sum_g 2l_g$. Using these notations, $\theta\mu_g$ is

$$\theta\mu_g = \frac{\frac{1}{L}\rho \sum_g (a_g + b_g) l_g}{\text{Denom}} \frac{(a_g + b_g)}{\frac{1}{L}\sum_g (a_g + b_g) l_g} = \frac{\rho(a_g + b_g)}{\text{Denom}}.$$

Plugging this formula of $\theta\mu_g$ and $1 - \theta = \frac{(1-\rho)\sum_g 2l_g}{\text{Denom}}$ in equation (17), we get

$$\begin{aligned} \text{bulk BAF} &= \frac{\rho(a_g + b_g) p_g + 0.5 \frac{1}{L}(1-\rho)\sum_g 2l_g}{\rho(a_g + b_g) + \frac{1}{L}(1-\rho)\sum_g 2l_g} \\ &= \frac{\rho(a_g + b_g) p_g + (1-\rho)}{\rho(a_g + b_g) + 2(1-\rho)} \\ &= \frac{\rho(a_g + b_g)\frac{b_g}{a_g+b_g} + (1-\rho)}{\rho(a_g + b_g) + 2(1-\rho)} \\ &= \frac{\rho b_g + (1-\rho)}{\rho(a_g + b_g) + 2(1-\rho)} \end{aligned}$$

which is the same as equation (16). □

However, Property (1) does not hold in SRT data. SRT measures gene expression, and the total expression in tumor and normal cells is not proportional to their numbers of genomic base pairs. We cannot derive an expression of tumor UMI proportion using tumor purity. Therefore, we use bulk BAF formula (17) with tumor UMI proportion θ.

S18 Evaluating likelihoods for pooled spots and individual spots

We justify aggregating spots of each clone in inferring the HMM model by relaxing the distribution to Poisson and Binomial distribution and proving that aggregation does not alter the optima in the HMM model.

Theorem 2. *If we simplifying the distribution of* $\mathbf{X}$ *to Poisson distributions and* $\mathbf{Y}$ *to Binomial distributions,*

$$X_{g,n} \sim Pois(T_n \mu_g^m)$$
$$Y_{g,n} \sim B(D_{g,n}, p_g^m, (1-p_g^m)),$$

then the optima of HMM objective of individual spots are the same as that of pseudobulk.

$$\arg\max_{\boldsymbol{\mu}^m, \mathbf{p}^m} \sum_{\mathbf{Z}_{\cdot,m}} \mathbb{P}(\mathbf{X}_{\cdot,I_m}, \mathbf{Y}_{\cdot,I_m}, \mathbf{D}_{\cdot,I_m} \mid \mathbf{Z}_{\cdot,m}, \boldsymbol{\mu}^m, \mathbf{p}^m))\mathbb{P}(\mathbf{Z}_{\cdot,m})$$
$$= \arg\max_{\boldsymbol{\mu}^m, \mathbf{p}^m} \sum_{\mathbf{Z}_{\cdot,m}} \mathbb{P}\Big(\sum_{n\in I_m} \mathbf{X}_{\cdot,n}, \sum_{n\in I_m} \mathbf{Y}_{\cdot,n}, \sum_{n\in I_m} \mathbf{D}_{\cdot,n} \mid \mathbf{Z}_{\cdot,m}, \boldsymbol{\mu}^m, \mathbf{p}^m)\Big)\mathbb{P}(\mathbf{Z}_{\cdot,m})$$

Proof. We first show that the likelihoods of pooled and individual spots are different by a constant scalar. Without loss of generality, we assume $\mathbf{Z}_{g,m} = k$ at bin g in clone m, the likelihood of individual spots at a bin g can be transformed as follows

$$\begin{aligned}
&\mathbb{P}(\mathbf{X}_{g,I_m}, \mathbf{Y}_{g,I_m}, \mathbf{D}_{g,I_m} \mid \mathbf{Z}_{\cdot,m}, \boldsymbol{\mu}^m, \mathbf{p}^m) \\
=& \prod_{n\in I_m} \mathbb{P}(X_{g,n} \mid Z_{g,m} = k, \mu_k^m)\mathbb{P}(Y_{g,n} \mid Z_{g,m} = k, D_{g,m}, p_k^m) \\
=& \prod_{n\in I_m} \frac{(T_n \mu_k^m)^{X_{g,n}} e^{-T_n \mu_k^m}}{X_{g,n}!} \binom{D_{g,n}}{Y_{g,n}} (p_g^m)^{Y_{g,n}} (1-p_g^m)^{D_{g,n}-Y_{g,n}} \\
=& \left(\prod_{n\in I_m} \frac{T_n^{X_{g,n}}}{X_{g,n}!} \binom{D_{g,n}}{Y_{g,n}}\right) (\mu_k^m)^{\sum_{n\in I_m} X_{g,n}} (p_k^m)^{\sum_{n\in I_m} Y_{g,n}} (1-p_k^m)^{\sum_{n\in I_m} D_{g,n}-Y_{g,n}} \\
=& const \times \frac{(\mu_k^m)^{\sum_{n\in I_m} X_{g,n}} e^{\mu_k^m \sum_{n\in I_m} T_n}}{(\sum_{n\in I_m} X_{g,n})!} \binom{\sum_{n\in I_m} D_{g,n}}{\sum_{n\in I_m} Y_{g,n}} (p_k^m)^{\sum_{n\in I_m} Y_{g,n}} (1-p_k^m)^{\sum_{n\in I_m} D_{g,n}-Y_{g,n}} \\
=& const \times \mathbb{P}(\sum_{n\in I_m} X_{g,n}, \sum_{n\in I_m} Y_{g,n}, \sum_{n\in I_m} D_{g,n} \mid \mathbf{Z}_{\cdot,m}, \boldsymbol{\mu}^m, \mathbf{p}^m).
\end{aligned}$$

Using the equality at each bin g, we can rewrite the HMM objective as

$$\begin{aligned}
&\arg\max_{\boldsymbol{\mu}^m, \mathbf{p}^m} \sum_{\mathbf{Z}_{\cdot,m}} \mathbb{P}(\mathbf{X}_{\cdot,I_m}, \mathbf{Y}_{\cdot,I_m}, \mathbf{D}_{\cdot,I_m} \mid \mathbf{Z}_{\cdot,m}, \boldsymbol{\mu}^m, \mathbf{p}^m))\mathbb{P}(\mathbf{Z}_{\cdot,m}) \\
=& \arg\max_{\boldsymbol{\mu}^m, \mathbf{p}^m} \sum_{\mathbf{Z}_{\cdot,m}} \prod_{g=1}^{G} \mathbb{P}(\mathbf{X}_{g,I_m}, \mathbf{Y}_{g,I_m}, \mathbf{D}_{g,I_m} \mid \mathbf{Z}_{\cdot,m}, \boldsymbol{\mu}^m, \mathbf{p}^m))\mathbb{P}(\mathbf{Z}_{\cdot,m}) \\
=& \arg\max_{\boldsymbol{\mu}^m, \mathbf{p}^m} const \times \left\{ \sum_{\mathbf{Z}_{\cdot,m}} \prod_{g=1}^{G} \mathbb{P}(\sum_{n\in I_m} X_{g,n}, \sum_{n\in I_m} Y_{g,n}, \sum_{n\in I_m} D_{g,n} \mid \mathbf{Z}_{\cdot,m}, \boldsymbol{\mu}^m, \mathbf{p}^m))\mathbb{P}(\mathbf{Z}_{\cdot,m}) \right\} \\
=& \arg\max_{\boldsymbol{\mu}^m, \mathbf{p}^m} const \times \left\{ \sum_{\mathbf{Z}_{\cdot,m}} \mathbb{P}(\sum_{n\in I_m} \mathbf{X}_{\cdot,n}, \sum_{n\in I_m} \mathbf{Y}_{\cdot,n}, \sum_{n\in I_m} \mathbf{D}_{\cdot,n} \mid \mathbf{Z}_{\cdot,m}, \boldsymbol{\mu}^m, \mathbf{p}^m))\mathbb{P}(\mathbf{Z}_{\cdot,m}) \right\}
\end{aligned}$$

□

The above derivation assumes that μ_k^n in Poisson distribution and p_k^m in Binomial distribution are shared across all spots. But in the mixture of clone cases, the parameters are unique to each spot if the mixing

proportions $\theta_{m,n}$ are distinct across spots.

$$X_{g,n} \sim Pois(T_n \sum_m \theta_{m,n} \mu_g^m)$$

$$Y_{g,n} \sim B(D_{g,n}, \sum_m \theta_{m,n} p_g^m, \sum_m \theta_{m,n}(1 - p_g^m))$$

But with the given mixing proportion $\theta_{m,n}$, we can pool a subset of spots with similar mixing proportion (and with high tumor proportion) and infer the HMM model only using the pooled subset.

Supplementary Figures and Tables

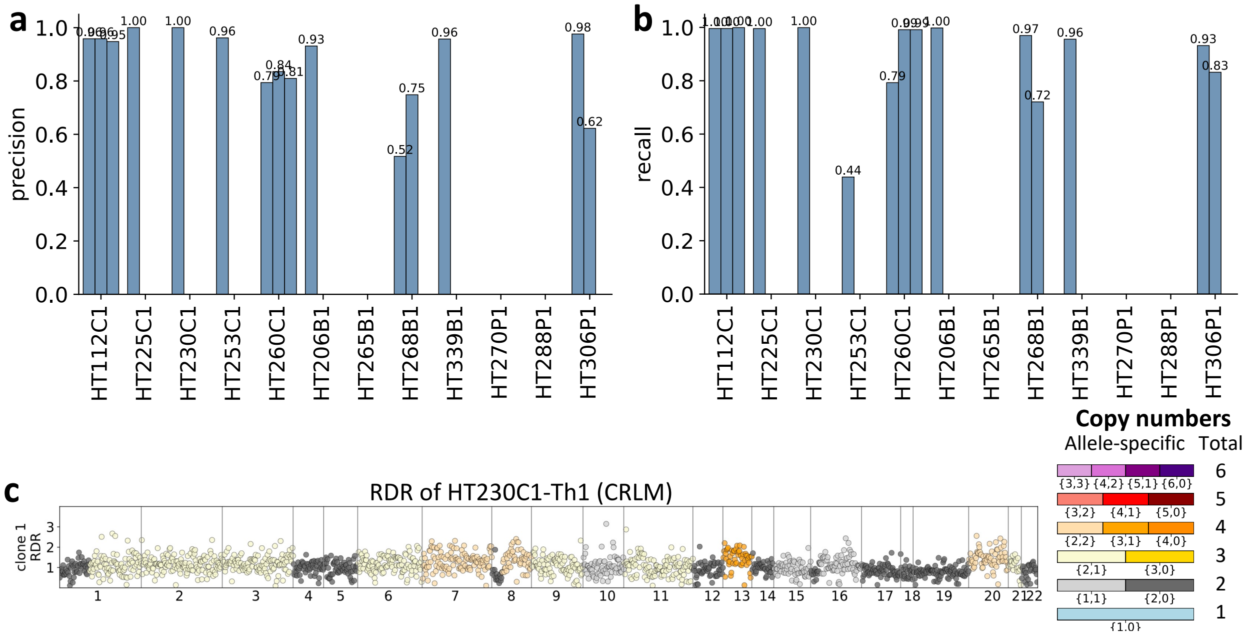

Figure 1: (a–b) Precision (a) and recall (b) of CalicoST-predicted aberrations compared with CNAs inferred by HATCHet2 from WES data. (c) RDR of a CRC liver metastasis patient HT230C1-Th1 with a triploid genome.

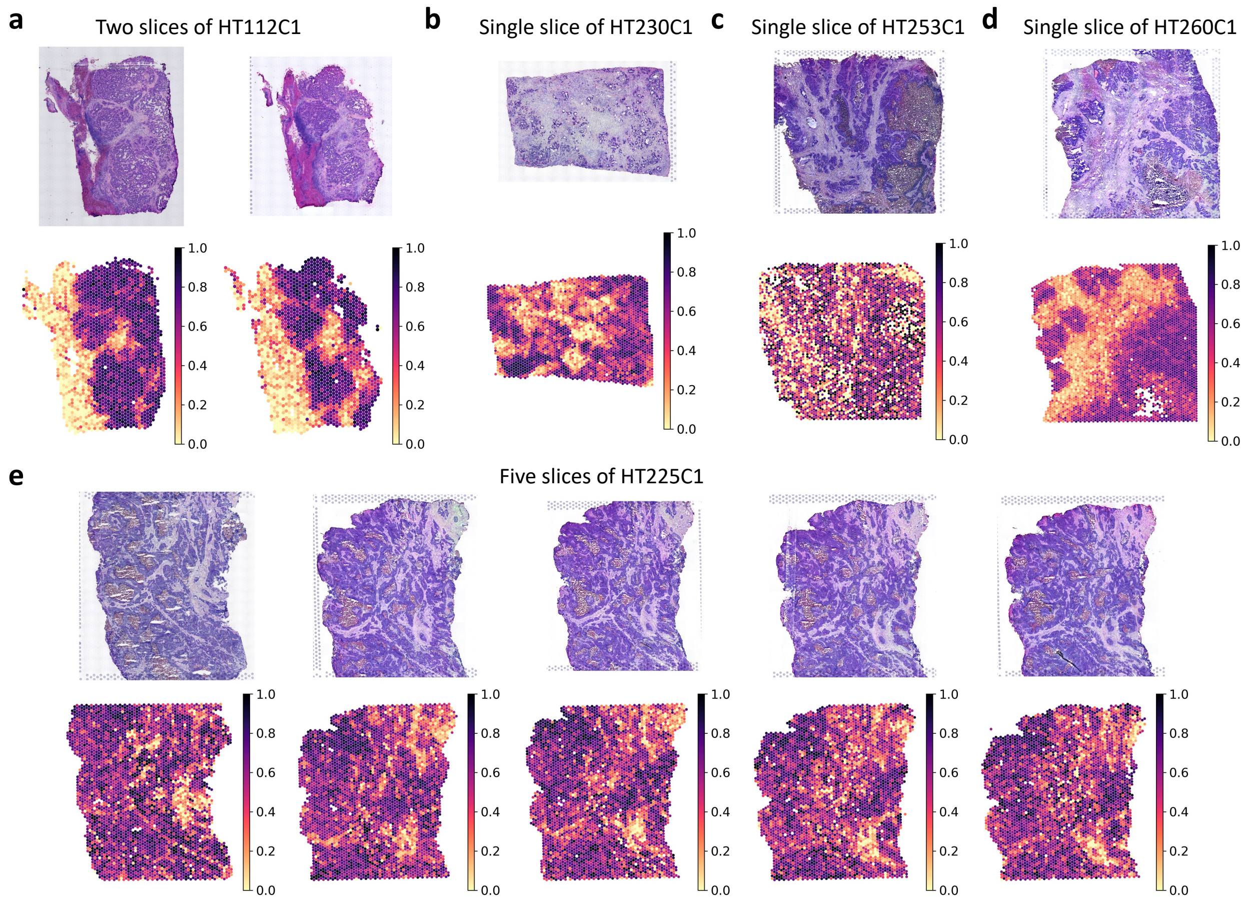

Figure 2: H&E images (top) and CalicoST-inferred tumor proportions (bottom) for CRC liver metastasis cancer samples: (a) HT112C1, (b) HT230C1, (c)HT253C1, (d) HT260C1, (e) HT225C1.

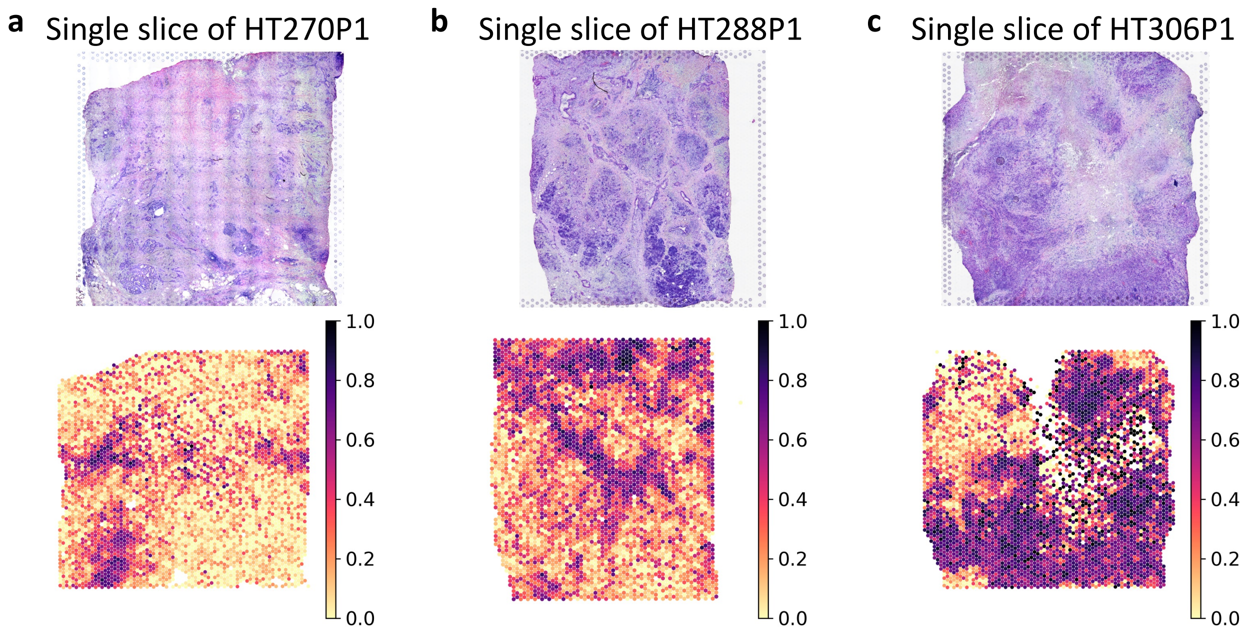

Figure 3: H&E images (top) and CalicoST-inferred tumor proportions (bottom) for PDAC samples: (a) HT270P1, (b) HT288P1, (c) HT306P1.

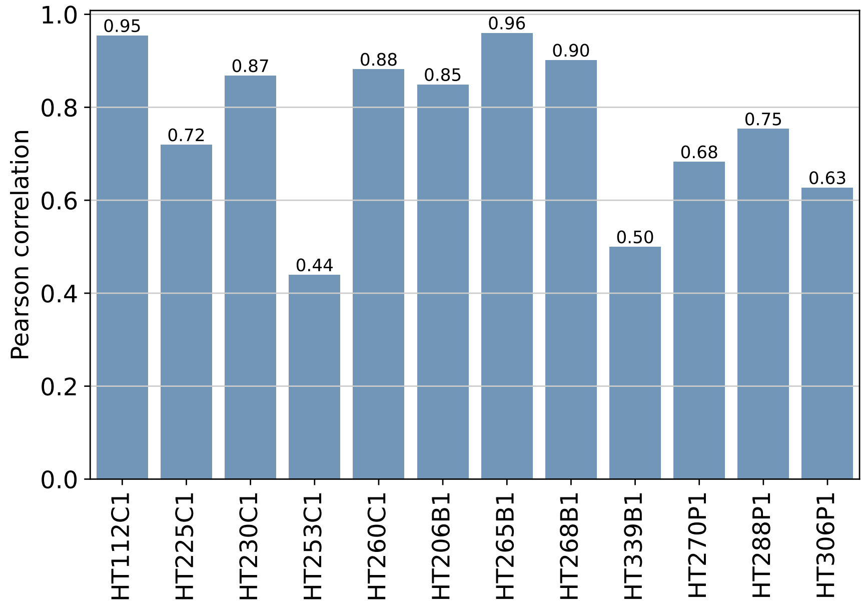

Figure 4: Pearson correlation between tumor proportions inferred by CalicoST using LOH events and tumor proportions inferred by deconvolution of gene expression using RCTD and matched scRNA-seq data from twelve HTAN samples.

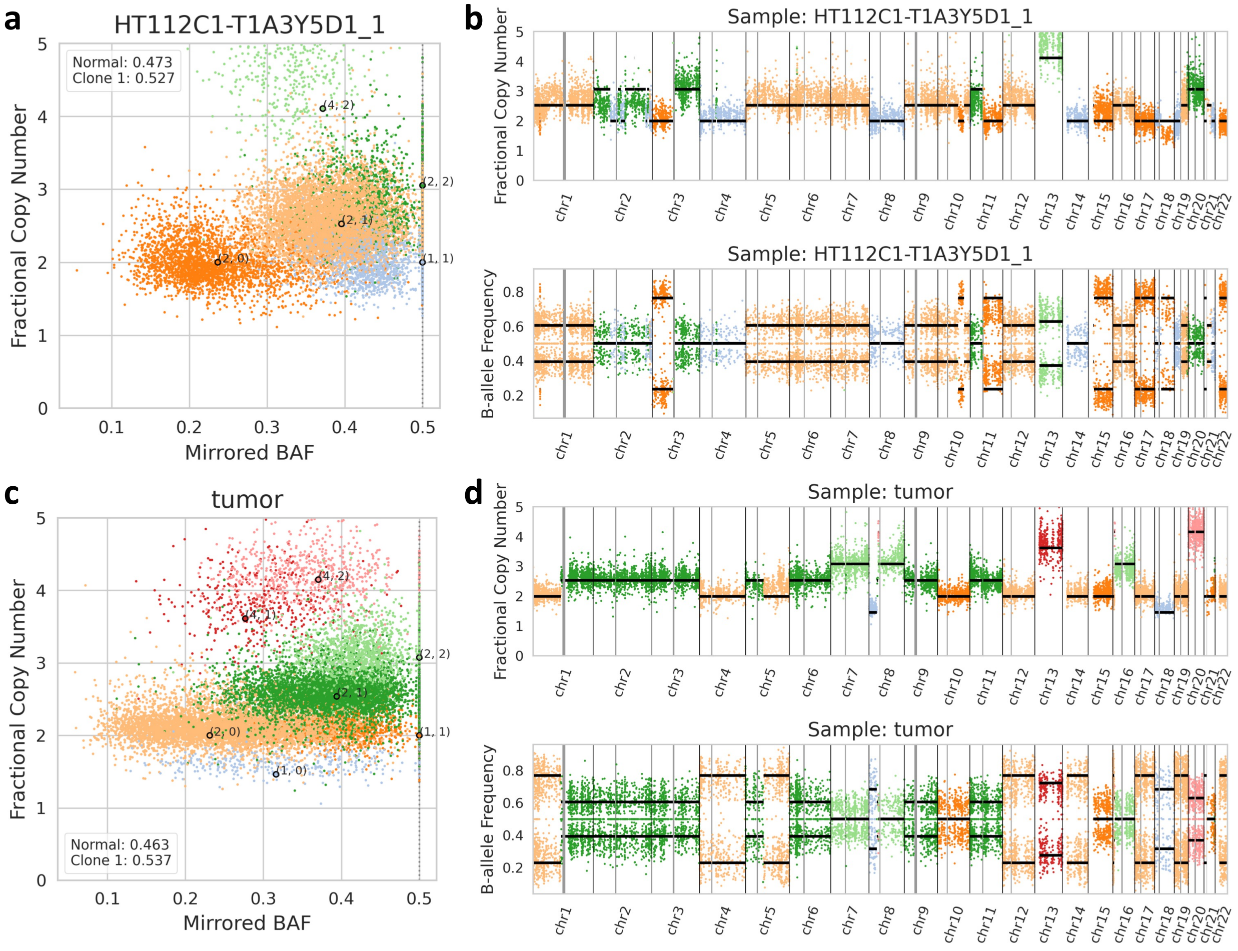

Figure 5: Allele-specific copy numbers inferred by HATCHet2 from WES data of patient HT112C1 (a–b) and HT230C1 (c–d). (a) The mirrored BAF (x-axis) and fractional copy number (y-axis) of each genomic bin in the WES data from sample HT112C1. Fractional copy number is proportional to RDR. Each color indicates an allele-specific copy number where the label $(\cdot, \cdot)$ indicates the integer copy numbers. (b) The fractional copy number and mirrored BAF and along the genome for the WES data of HT112C1. Each color indicates the allele-specific copy number under the same color scheme as (a). (c–d) The same plots for the HT230C1 WES sample.

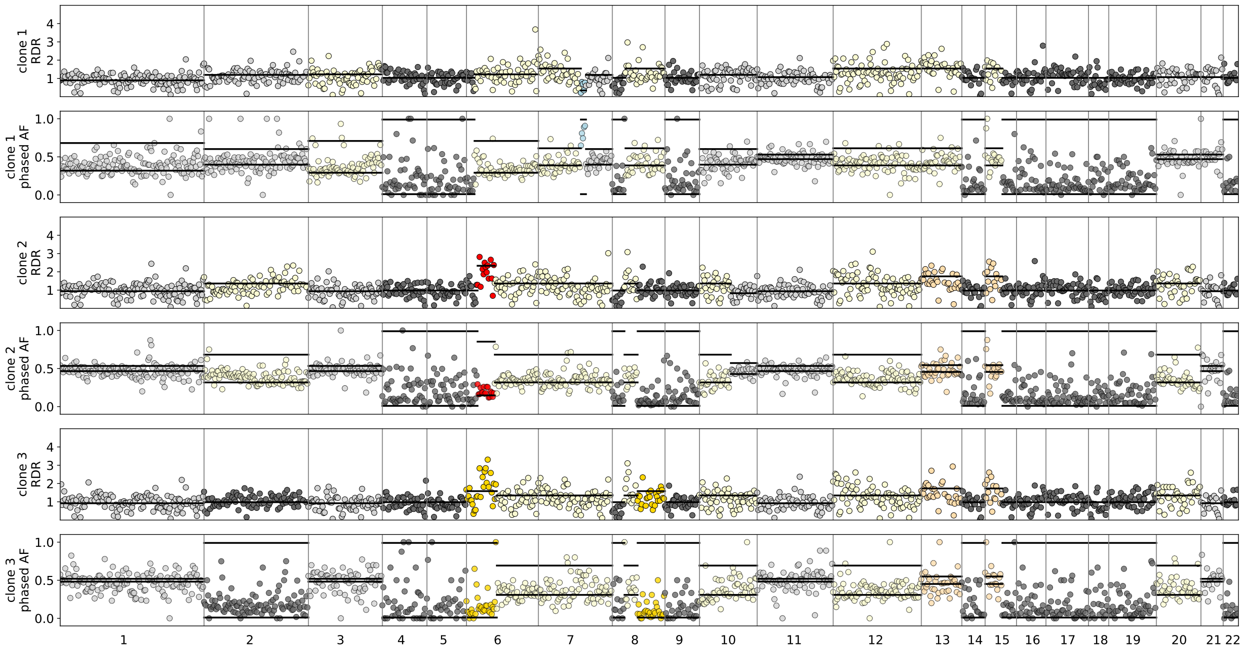

Figure 6: Observed RDR and BAF values for each inferred clone in sample HT260C1.

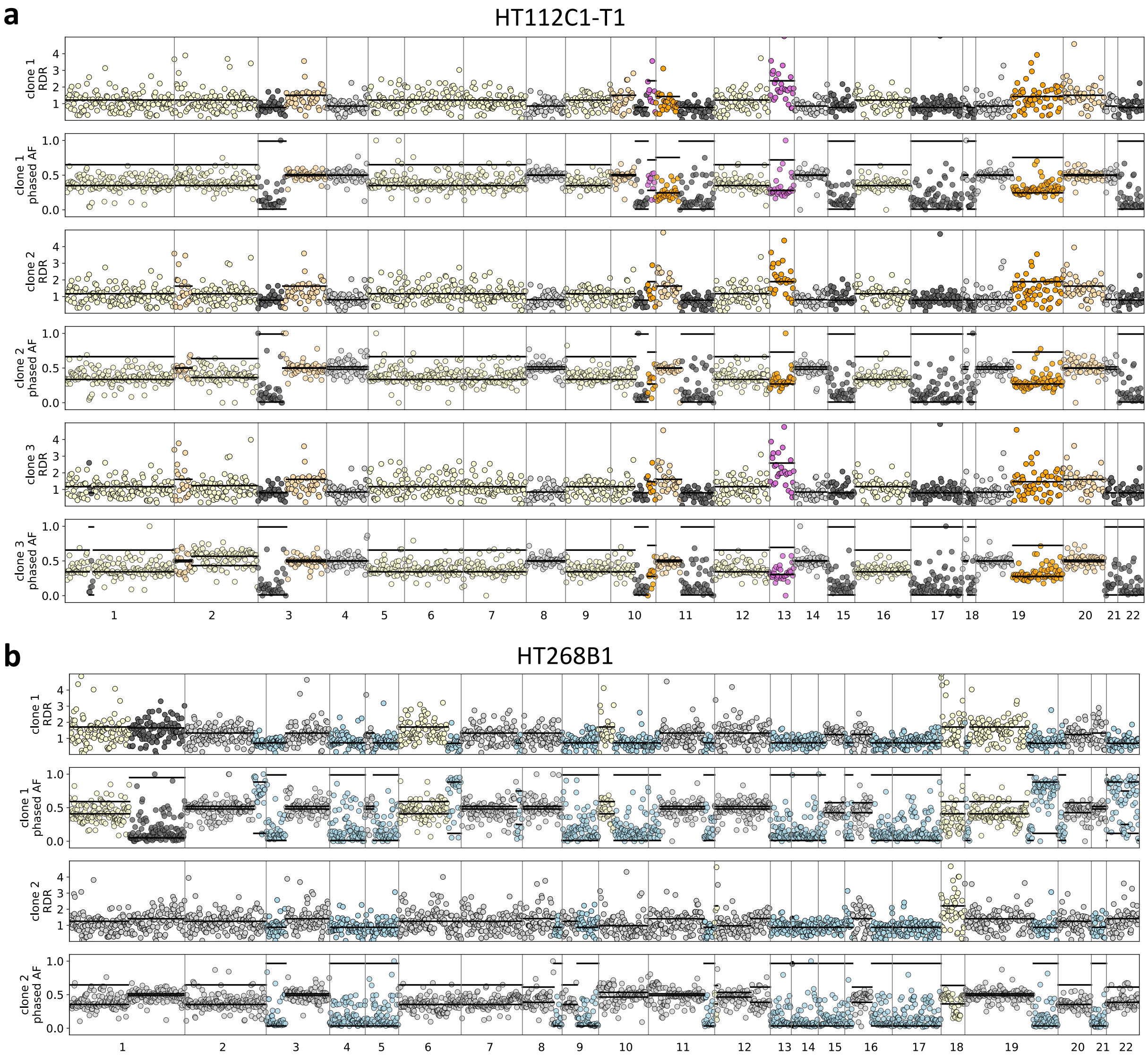

Figure 7: RDR and BAF for each inferred cancer clone of a CRC liver metastasis patient HT112C1 (a) and a breast cancer patient HT268B1. (b). x-axis indicates the coordinates along the genome. Color scheme is the same as Fig 4b,d.

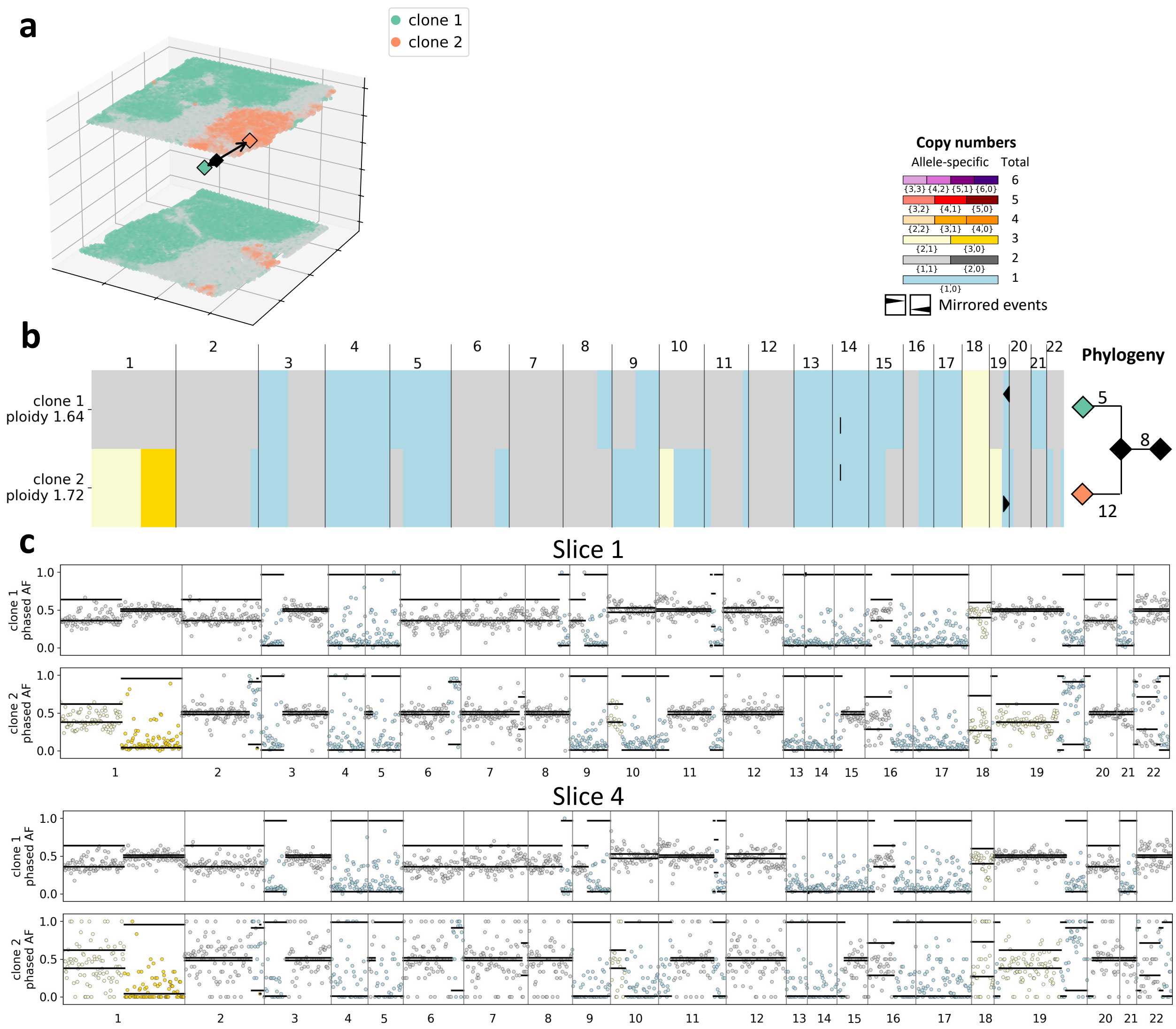

Figure 8: Clones and copy number profiles inferred by CalicoST on the distance pair of distant slices (slice 1 and slice 4) from HT268B1 breast cancer sample. (a) CalicoST-inferred cancer clones. Grey scale indicates the tumor proportion and other colors distinguish inferred clones. (b) Allele-specific copy numbers inferred by CalicoST and the reconstructed phylogenetic tree. (c) BAF along the genome of each clone in slice 1 (top) and slice 4 (bottom).

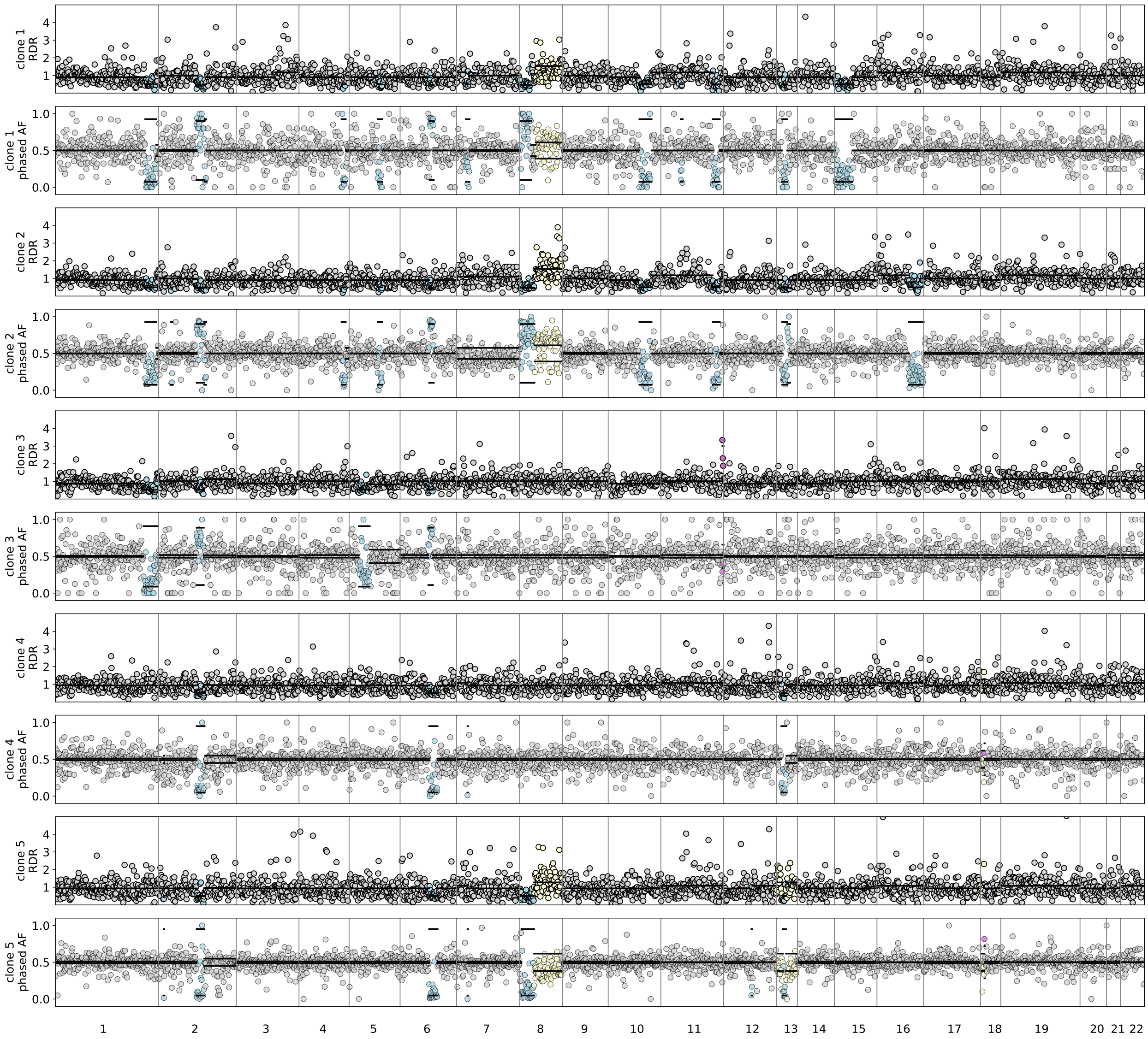

Figure 9: Observed RDR and BAF values for each inferred clone in the prostate cancer samples.

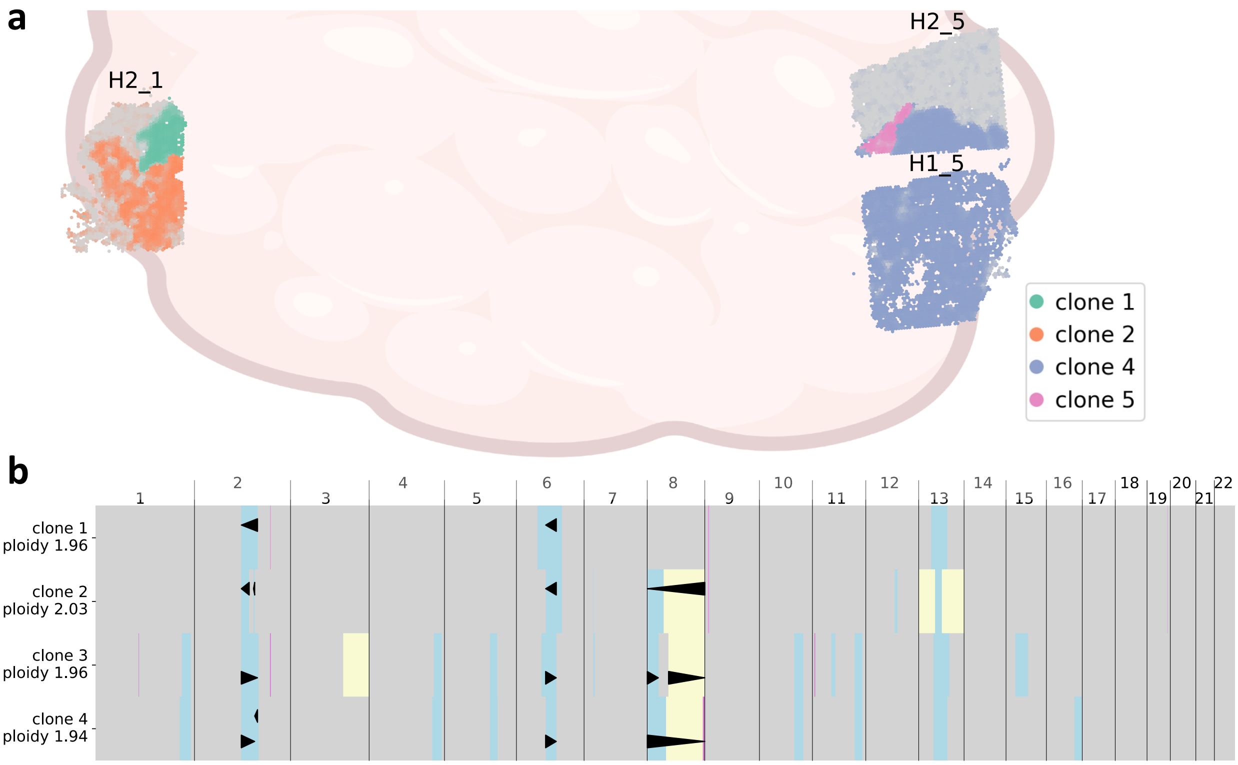

Figure 10: Cancer clones and copy number profiles inferred by CalicoST-on a subset of three slices (H2_1, H1_5, H2_5) of the multi-section prostate cancer dataset. The inferred clones using three slices have an ARI of 0.956 compared to clones inferred by CalicoST on all five slices. Moreover, the CNA profiles of the four clones have 95.84%, 98.36%, 99.06%, and 99.18% exact match with the CNAs inferred using all slices, respectively.

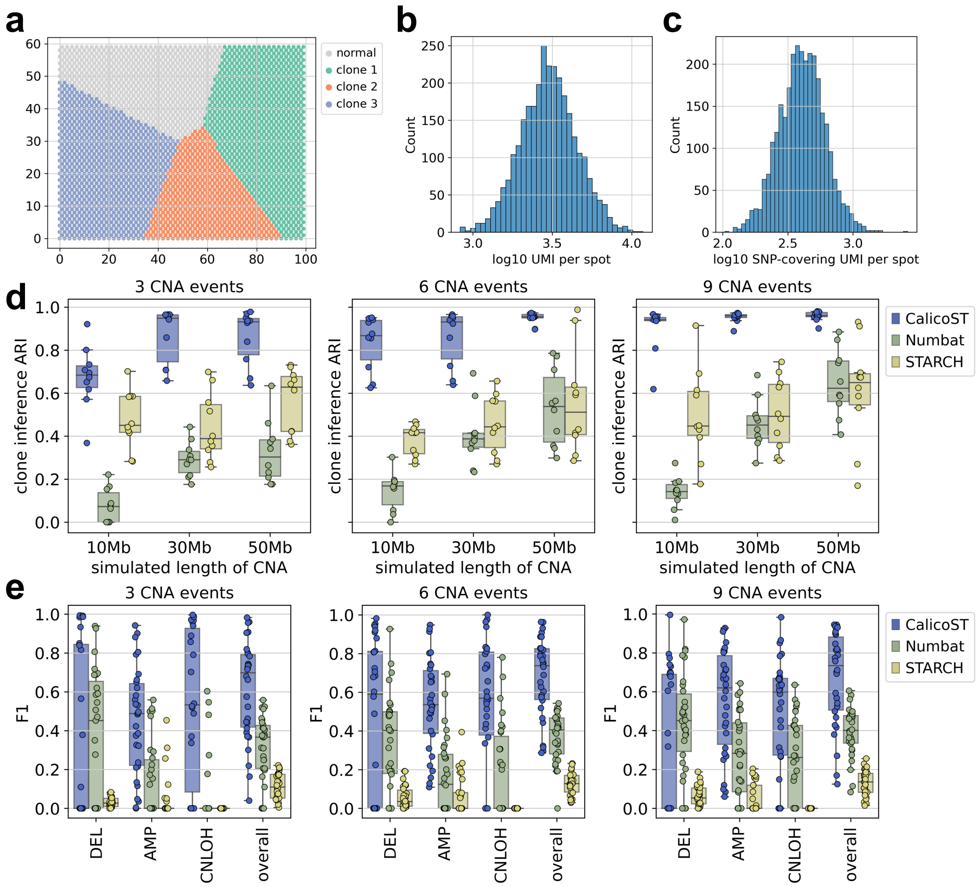

Figure 11: Accuracy of clone and CNA inference on simulated data. (a) Spatial distribution of normal regions and cancer clones in one simulation. (b) Histogram of log10 UMI count per spot on simulated data. (c) Histogram of log10 SNP-covering UMI per spot on simulated data. (d) Accuracy of inferred tumor clones – computed using the adjusted Rand index (ARI) for CalicoST, STARCH, and Numbat. Each point in the plot represents a replicate with the corresponding number and length of CNAs. Each box contains the ARI of ten simulated samples with the CNA lengths indicated by the x-axis and number of events indicated in the subplot title. (e) F1 score of identifying genes affected by deletions (DEL), amplifications (AMP), CNLOH, and all three events by the three methods. Each point in the stripplot represents a simulated sample. Each box plot contains data from 30 simulated samples with the number of CNA events indicated by the subplot title. The upper and lower bounds of the box denote the 25% and 75% quantile, the center line denotes the median, and the lower (higher) whiskers denote smallest (largest) value that within the 1.5 times of IQR (Interquartile Range).

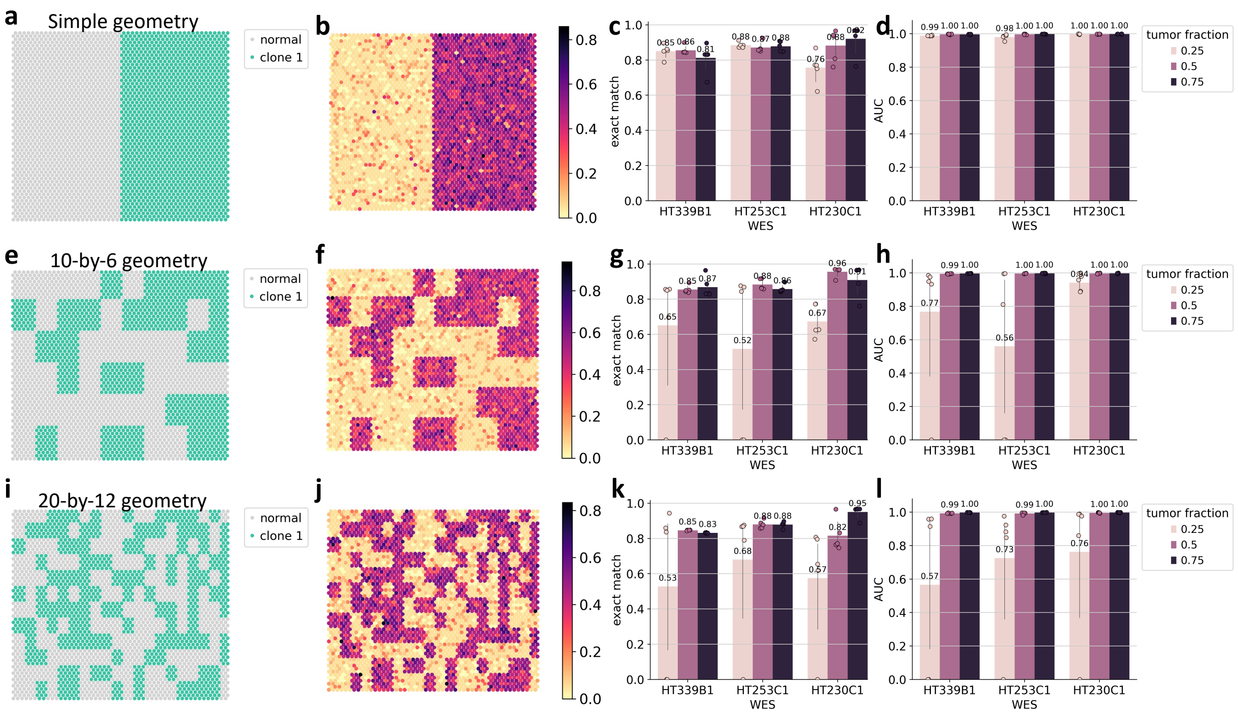

Figure 12: (a) Simulated spatial organization of tumor and normal spots under a simple geometry with 50% tumor spots. (b) Tumor proportion per spot inferred by CalicoST of the simulated data based on WES of HT230C1. (c) Accuracy of the CNAs inferred by CalicoST evaluated by the exact match with HATCHet2 copy numbers. (d) Accuracy of the tumor proportions inferred by CalicoST evaluated by the AUC of predicted tumor spots. (e–h) Simulated spatial organization with a partition of space into 10-by-6 blocks, CalicoST-inferred tumor proportions based on WES of HT230C1, and the accuracy of CalicoST-inferred CNAs and tumor proportions. (i–l) Simulated spatial organization with a partition of space into 20-by-12 blocks, CalicoST-inferred tumor proportions based on WES of HT230C1, and the accuracy of CalicoST-inferred CNAs and tumor proportions. For (c–d), (g–h), and (k–l), each bar contains five replicates in the simulation, which are conducted under the specified geometry, with the tumor fraction indicated by the bar color, and WES sample indicated by the x-axis. The error bar denotes the 95% confidence interval constructed by a bootstrap of the distribution provided by the seaborn python package [17].

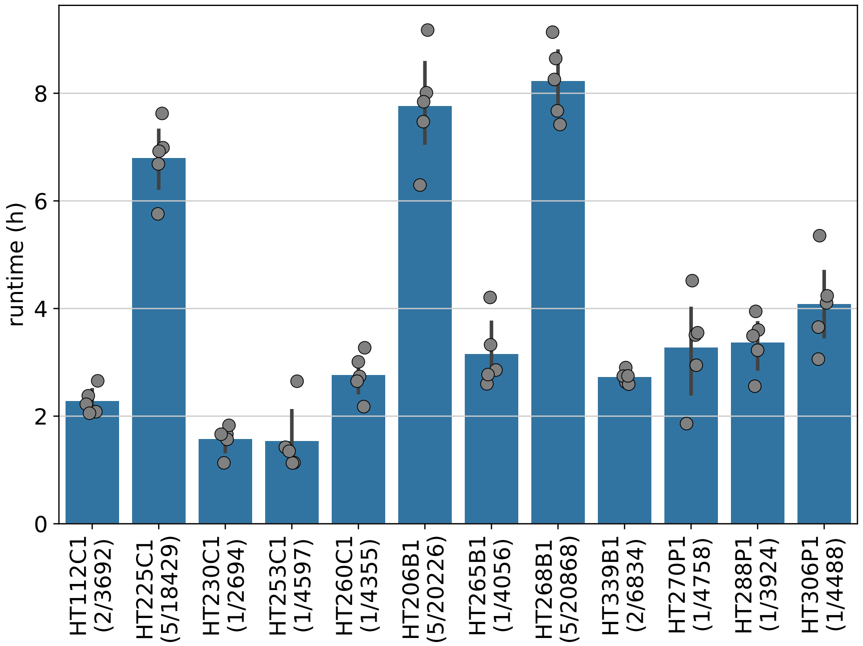

Figure 13: Runtime of CalicoST in hours for the twelve HTAN patients with (m/n) indicating the number slices and total number of spots for each patient. Error bars are for ten random initializations of CalicoST clone labels. The error bar denotes the 95% confidence interval constructed by a bootstrap of the distribution provided by the seaborn package.

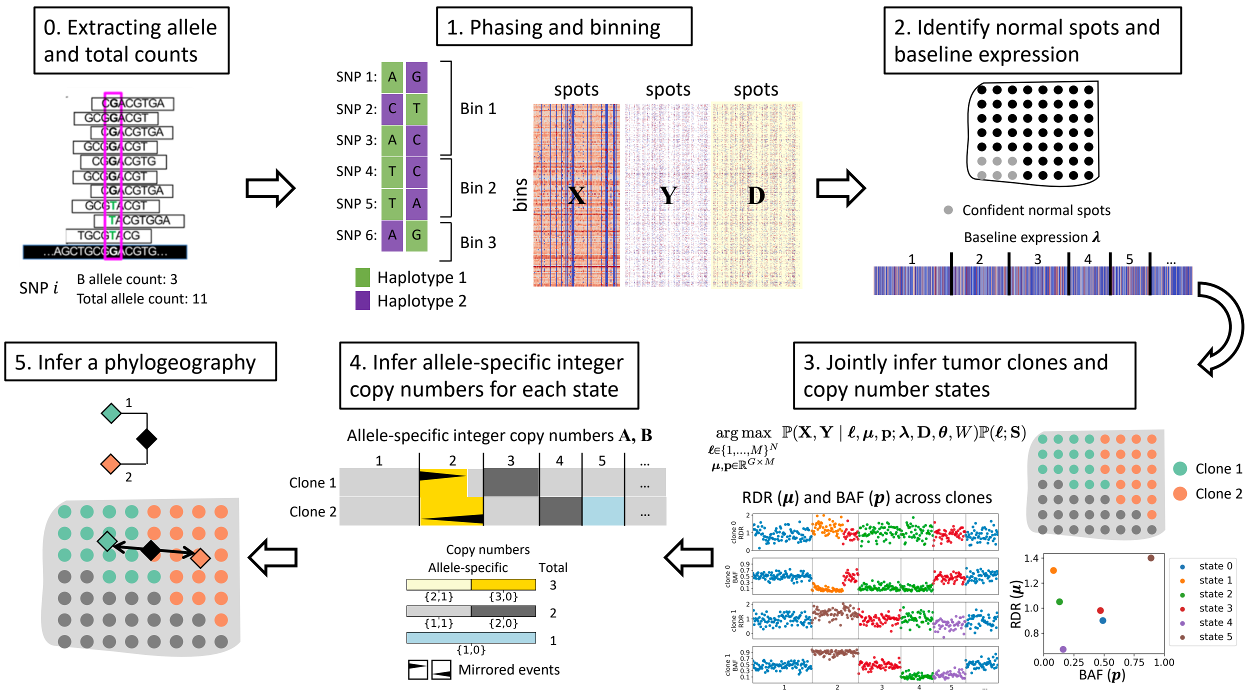

Figure 14: **Workflow of CalicoST.** In step 0, CalicoST extracts the B allele and total counts of heterozygous SNP loci from the BAM file to distinguish between two alleles. In step 1, CalicoST phases the SNPs and aggregates transcript counts and allele counts of each haplotype along the genome. In step 2, CalicoST identifies normal spots. In step 3, jointly infers tumor clones and estimates a latent RDR and BAF value for each copy number state across all involved genomic bins in each clone. In step 4, CalicoST infers allele-specific integer copy numbers using the latent RDR and BAF values. In step 5, CalicoST reconstructs a phylogeographic model of tumor evolution by inferring a tumor phylogeny using LOH events and inferring spatial locations of ancestral clones.

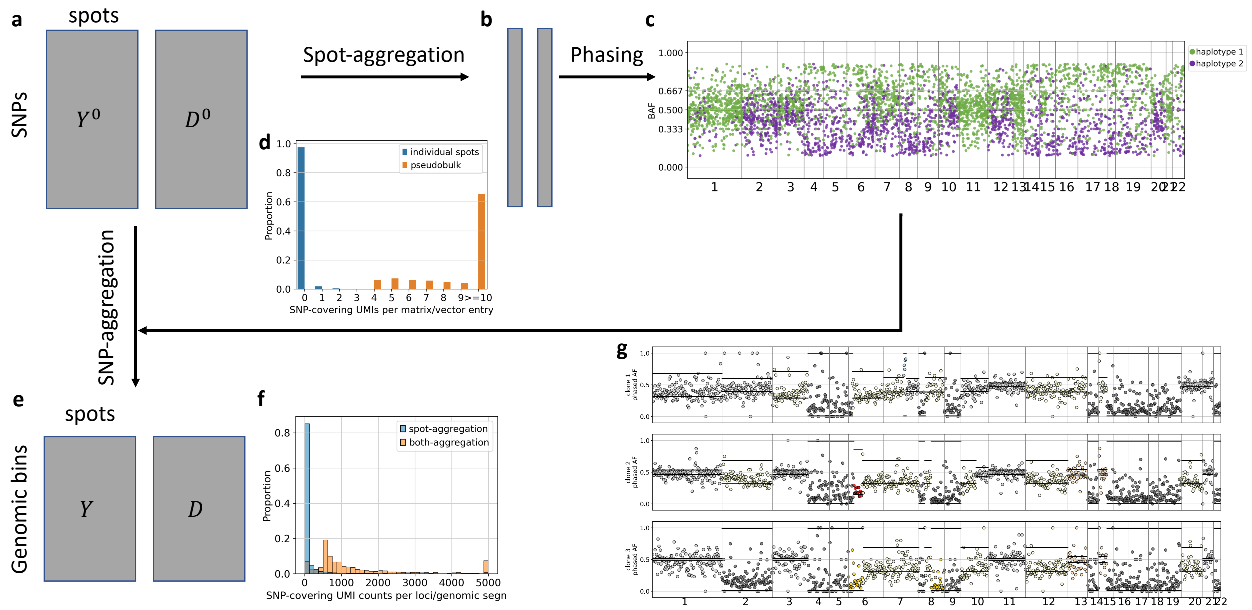

Figure 15: The phasing and binning pipeline of CalicoST. (a) The input is the SNP-by-spot allele count matrix Y^0 of the haplotype 1 inferred by population-based phasing and total allele count matrix D^0. (b) CalicoST attempts to correct potential errors in population-based phasing by modeling the observed BAF in a pseudobulk sample containing all spots of spots using an Hidden Markov Model (HMM) whose hidden states correspond to mirrored allele-specific copy numbers. (c) The output of phasing on HTAN sample HT260C1. Each point represents a SNP and is colored by the haplotype label inferred by the HMM model. (d) Histogram of total allele counts (entries in D^0) in individual spots and in pseudobulk of HT260C1.(e) CalicoST calculates a genomic bin-by-spot allele count matrices Y of haplotype 1 and total allele count matrix D by aggregating the allele counts D^0 and D^0 across adjacent SNPs in the genome using the haplotype inferred from the pseudobulk per SNP obtained in (c). (f) Total allele count of HTAN sample HT260C1 for each SNP or genomic bin in pseudobulk. (g) BAF for each genomic bin for the two inferred clones in sample HT260C1.

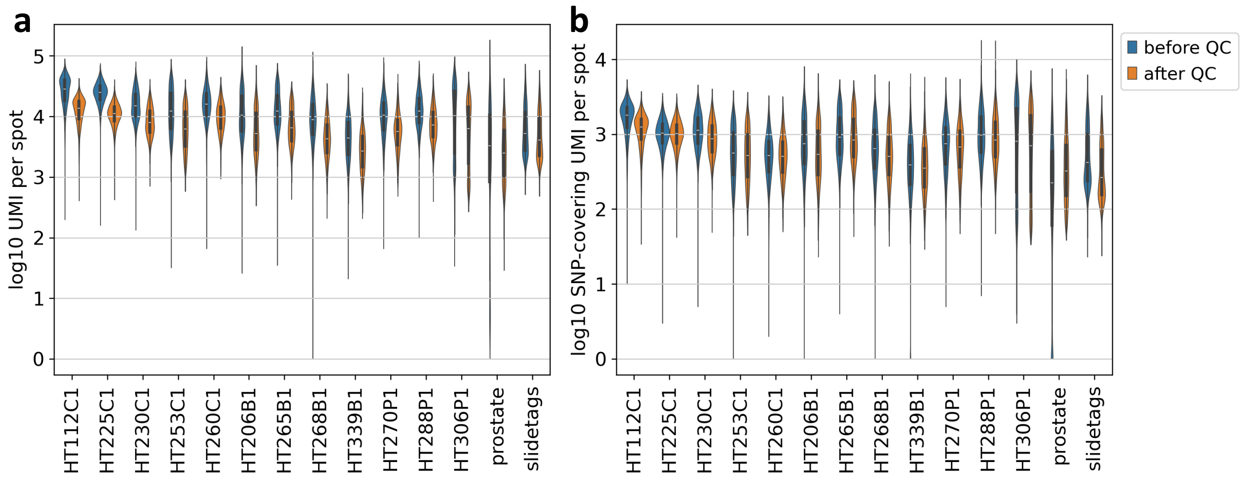

Figure 16: (a) Distribution of UMIs per spot (log10 scale) for each patient. (b) Distribution of SNP-covering UMIs (log10 scale) per spot for each patient. "QC" refers to the filtering of spots, genes, and SNPs by CalicoST. The number of spots included in each violin before QC is indicated by the "Total spots" column in Table 2, which are 3702, 18457, 2697, 4617, 4405, 20317, 4065, 21154, 7084, 4770, 3928, 4779, 17372, and 2535 from left to right. The number of spots included in each violin after QC is indicated by "Total spots (after QC)" column in Table 2, which are 3692, 18429, 2694, 4597, 4355, 20226, 4056, 20868, 6834, 4758, 3924, 4488, 13311, and 2527 from left to right. The upper and lower bounds of the box inside each violin denote the 25% and 75% quantile, the center denotes the median. The range of each violin denotes the range of the data, and the width denotes the density of data points.

Table 2: Summary statistics of datasets analyzed by CalicoST. "after QC" refers to the filtering of spots, genes, and SNPs by CalicoST.

Patient	Cancer type	Number slices	Total spots	Total spots (after QC)
HT112C1	CRC liver metastasis	2	3702	3692
HT225C1	CRC liver metastasis	5	18457	18429
HT230C1	CRC liver metastasis	1	2697	2694
HT253C1	CRC liver metastasis	1	4617	4597
HT260C1	CRC liver metastasis	1	4405	4355
HT206B1	Breast cancer	5	20317	20226
HT265B1	Breast cancer	1	4065	4056
HT268B1	Breast cancer	5	21154	20868
HT339B1	Breast cancer	2	7084	6834
HT270P1	PDAC	1	4770	4758
HT288P1	PDAC	1	3928	3924
HT306P1	PDAC	1	4779	4488
Multi-section prostate	Prostate cancer	5	17372	13311
Slide-tags	Melanoma	1	2535	2527

Table 3: Summary statistics for genomic bins constructed by CalicoST for each patient, including the number of bins along the genome, median bin length in kb, median number of SNPs per bin, median number of expressed genes per bin, and median SNP-covering UMIs per bin per spot.

Patient	Number bins	Median bin length (kb)	Median num SNPs per bin	Median number expressed genes per bin	Median SNP-covering UMIs per bin per spot
HT112C1	1558	757.36	7	9	0.27
HT225C1	2503	324.92	8	5	0.09
HT230C1	1415	934.92	7	10	0.33
HT253C1	1239	1164.63	7	10	0.19
HT260C1	1212	1268.09	8	11	0.20
HT206B1	2475	375.91	10	5	0.07
HT265B1	1862	590.33	7	7	0.24
HT268B1	2451	376.22	8	5	0.07
HT339B1	1574	719.91	7	8	0.12
HT270P1	1511	798.45	8	9	0.18
HT288P1	1567	723.56	8	9	0.23
HT306P1	1482	786.72	7	9	0.25
Multi-section prostate cancer	2697	359.21	13	4	0.05
Slide-tags	1721	918.51	60	8	0.21

Table 4: Summary statistics for deletions identified by CalicoST in clone 1 of the multi-section prostate cancer patient.

CHR	START	END	clone	length (kb)	num_bins	B allele count	total allele count	A/B copy
1	212363931	237833788	clone1	25,469.86	26	115	995	(0,1)
2	117814691	156591210	clone1	38,776.52	19	775	903	(1,0)
2	157257705	162267542	clone1	5,009.84	4	16	132	(0,1)
4	163110073	176331856	clone1	13,221.78	10	65	405	(0,1)
5	115124762	132963411	clone1	17,838.65	11	70	551	(0,1)
6	73695785	99558095	clone1	25,862.31	10	2451	2629	(1,0)
7	16791811	29916764	clone1	13,124.95	9	2438	52265	(0,1)
8	232137	27810203	clone1	27,578.07	27	1041	1272	(1,0)
10	87050202	109897191	clone1	22,846.99	29	170	972	(0,1)
11	47354860	49206069	clone1	1,851.21	5	35	376	(0,1)
11	106674012	125098284	clone1	18,424.27	17	57	491	(0,1)
13	35768652	67230101	clone1	31,461.45	12	189	1708	(0,1)
15	22983192	55196907	clone1	32,213.72	41	847	6261	(0,1)

Table 5: Evaluation of the classification of normal vs. cancer spots by CalicoST-inferred tumor proportions across twelve HTAN samples. The evaluation metric is the area under the ROC curve (AUC) compared with manual annotations from H&E images. For each sample, the third column gives the proportion of allele counts that are within genomic regions that CalicoST infers to be LOH.

patient	AUC	Proportion allele counts within LOH
HT112C1	0.950	0.105
HT225C1	0.870	0.042
HT230C1	0.937	0.225
HT253C1	0.623	0.017
HT260C1	0.871	0.228
HT206B1	0.925	0.323
HT265B1	0.969	0.262
HT268B1	0.959	0.252
HT339B1	0.811	0.110
HT270P1	0.845	0.130
HT288P1	0.849	0.113
HT306P1	0.622	0.045

References

[1] Teng Gao, Ruslan Soldatov, Hirak Sarkar, Adam Kurkiewicz, Evan Biederstedt, Po-Ru Loh, and Peter V Kharchenko. Haplotype-aware analysis of somatic copy number variations from single-cell transcriptomes. Nature Biotechnology, pages 1–10, 2022.

[2] Xianjie Huang and Yuanhua Huang. Cellsnp-lite: an efficient tool for genotyping single cells. Bioinformatics, 37(23):4569–4571, 2021.

[3] 1000 Genomes Project Consortium et al. A global reference for human genetic variation. Nature, 526(7571):68, 2015.

[4] Po-Ru Loh, Petr Danecek, Pier Francesco Palamara, Christian Fuchsberger, Yakir A Reshef, Hilary K Finucane, Sebastian Schoenherr, Lukas Forer, Shane McCarthy, Goncalo R. Abecasis, Richard Durbin, and Alkes L Price. Reference-based phasing using the Haplotype Reference Consortium panel. Nature Genetics, 48(11):1443–1448, nov 2016.

[5] Dylan M Cable, Evan Murray, Luli S Zou, Aleksandrina Goeva, Evan Z Macosko, Fei Chen, and Rafael A Irizarry. Robust decomposition of cell type mixtures in spatial transcriptomics. Nature Biotechnology, 40(4):517–526, 2022.

[6] Ying Ma and Xiang Zhou. Spatially informed cell-type deconvolution for spatial transcriptomics. Nature Biotechnology, pages 1–11, 2022.

[7] Julien Stoehr. A review on statistical inference methods for discrete markov random fields. arXiv preprint arXiv:1704.03331, 2017.

[8] Julian Besag. Spatial interaction and the statistical analysis of lattice systems. Journal of the Royal Statistical Society: Series B (Methodological), 36(2):192–225, 1974.

[9] Xinhao Liu, Ron Zeira, and Benjamin J Raphael. Partial alignment of multislice spatially resolved transcriptomics data. Genome Research, 33(7):1124–1132, 2023.

[10] Simone Zaccaria and Benjamin J Raphael. Accurate quantification of copy-number aberrations and whole-genome duplications in multi-sample tumor sequencing data. Nature Communications, 11(1):1–13, 2020.

[11] BOUAYAD AGHA Salima and MD Bellefon. Spatial autocorrelation indices, pages 60–62. 2018.

[12] Andrew JC Russell, Jackson A Weir, Naeem M Nadaf, Matthew Shabet, Vipin Kumar, Sandeep Kambhampati, Ruth Raichur, Giovanni J Marrero, Sophia Liu, Karol S Balderrama, et al. Slide-tags enables single-nucleus barcoding for multimodal spatial genomics. Nature, 625(7993):101–109, 2024.

[13] Andrew Erickson, Mengxiao He, Emelie Berglund, Maja Marklund, Reza Mirzazadeh, Niklas Schultz, Linda Kvastad, Alma Andersson, Ludvig Bergenstråhle, Joseph Bergenstråhle, et al. Spatially resolved clonal copy number alterations in benign and malignant tissue. Nature, 608(7922):360–367, 2022.

[14] Chia-Kuei Mo, Jingxian Liu, Siqi Chen, Erik Storrs, Andre Luiz Targino da Costa, Michael D. Iglesia, Cong Ma, Reyka G. Jayasinghe, Andrew Houston, John M. Herndon, Jacqueline Mudd, Xinhao Liu, Alla Karpova, Andrew Shinkle, Austin N. Southard-Smith, Michael C. Wendl, S. Peter Goedegebuure, Abdurrahman Taha Mousa Ali Abdelzaher, Peng Bo, Lauren Fulghum, Samantha Livingston, Metin Balaban, Angela Hill, Joseph E. Ippolito, Vesteinn Thorsson, Jason M. Held, Eric H. Kim, Peter O. Bayguinov, Albert H. Kim, Kooresh I. Shoghi, Sidharth V. Puram, Tao Ju, Melissa A. Reimers, Cody Weimholt, Liang-I Kang, Deborah J. Veis, Milan G. Chheda, Russell Pachynski, Katherine C. Fuh, William E. Gillanders, Ryan C. Fields, Benjamin J. Raphael, Feng Chen, and Li Ding. Spatial clonal evolution and clone-specific microenvironment interactions within three-dimensional tumor structures. Nature, 2023. Submitted.

[15] Yuchao Jiang, Yu Qiu, Andy J Minn, and Nancy R Zhang. Assessing intratumor heterogeneity and tracking longitudinal and spatial clonal evolutionary history by next-generation sequencing. Proceedings of the National Academy of Sciences, 113(37):E5528–E5537, 2016.

[16] Maxime Tarabichi, Adriana Salcedo, Amit G Deshwar, Máire Ni Leathlobhair, Jeff Wintersinger, David C Wedge, Peter Van Loo, Quaid D Morris, and Paul C Boutros. A practical guide to cancer subclonal reconstruction from dna sequencing. Nature Methods, 18(2):144–155, 2021.

[17] Michael L. Waskom. seaborn: statistical data visualization. Journal of Open Source Software, 6(60):3021, 2021.
